# Supplementary material for: Comparing and integrating human mobility data sources for measles transmission modeling in Zambia
Source: PLOS Glob Public Health. 2025 May 20;5(5):e0003906. doi: 10.1371/journal.pgph.0003906 (PMC12091742; doi:10.1371/journal.pgph.0003906)
Supplement: S1 Table — (DOCX) [file pgph.0003906.s001.docx]

S1 Table. Key features of datasets used to estimate population mobility in Zambia.

| **Dataset** | **Time period** | **Geographical scope** | **Individuals included** | **Informs departure / diffusion** | **Definition of travel indicator** |
| --- | --- | --- | --- | --- | --- |
| Mobile phone | March 1, 2020 – December 30, 2020 | 107 districts | Zamtel subscribers | Departure and diffusion | Change in district in which primary tower location is recorded on subsequent days |
| Facebook | April 1, 2020 – May 21, 2022 | 19 districts retained; 16 of these are districts with reported travel outside district, and 3 are destinations only | Individuals with Facebook application installed on their mobile device, that opted to share location | Departure and diffusion | Change in district in which GPS location is reported across two 8-hr periods |
| Travel survey | March – June 2022 | 2 districts (Choma and Ndola) | Respondents to community-based multi-stage cluster survey; respondents selected are random adults (15 years and over); caretakers of children 1 – 4 years old; and caretakers of children 5 – 14 years old | Departure and diffusion | Overnight travel out of district in the last 2 months |
| Demographic and Health Survey (DHS) | 17 July 2018 – 24 January 2019 | 112 districts | Respondents to community-based multi-stage cluster survey; women aged 15 – 49 years old, and men aged 15 – 59 years old | Departure only | Overnight travel in the last 12 months |
