# Supplementary material for: Comparing and integrating human mobility data sources for measles transmission modeling in Zambia
Source: PLOS Glob Public Health. 2025 May 20;5(5):e0003906. doi: 10.1371/journal.pgph.0003906 (PMC12091742; doi:10.1371/journal.pgph.0003906)
Supplement: S2 Table — Travel survey is excluded from this comparison given that it had information on travel from only two origin districts (Ndola and Choma Districts). (DOCX) [file pgph.0003906.s002.docx]

**S2 Table. Spearman’s rho correlation for probabilities of departure between three datasets used to quantify mobility, Zambia. Travel survey is excluded from this comparison given that it had information on travel from only two origin districts (Ndola and Choma Districts).**

| Pairs of datasets | Spearman’s correlation coefficient | p-value |
| --- | --- | --- |
| Facebook – Mobile phone | 0.529 | 0.045 |
| Mobile phone – DHS | 0.083 | 0.4028 |
| Facebook – DHS | -0.082 | 0.7629 |
