# Supplementary material for: Comparing and integrating human mobility data sources for measles transmission modeling in Zambia
Source: PLOS Glob Public Health. 2025 May 20;5(5):e0003906. doi: 10.1371/journal.pgph.0003906 (PMC12091742; doi:10.1371/journal.pgph.0003906)
Supplement: S3 Table — Cumulative infections show mean and 95% confidence interval values of cumulative infections over the course of simulation time (72 weeks). Peak infections are the mean and 95% confidence interval for the maximum number of infections in a single 2-week timestep. (DOCX) [file pgph.0003906.s003.docx]

S3 Table. Results of measles simulations, by the source of departure and diffusion mobility data. Cumulative infections show mean and 95% confidence interval values of cumulative infections over the course of simulation time (72 weeks). Peak infections are the mean and 95% confidence interval for the maximum number of infections in a single 2-week timestep.

| Dataset | | Cumulative infections | Peak infections | Districts with introduction (%) |
| --- | --- | --- | --- | --- |
| Departure | Diffusion |  |  |  |
| Mobile phone data | Mobile phone data | 2200 [1500; 3300] | 310 [210; 480] | 33 |
| Facebook | Facebook | 2500 [1700; 3700] | 350 [240; 510] | 42 |
| Travel survey | Travel survey | 2800 [1500; 4200] | 390 [220; 580] | 98 |
| *Mobile phone data diffusion* | |  |  |  |
| Weighted Mobile phone data | Mobile phone data | 2300 [1500; 3400] | 330 [200; 480] | 35 |
| DHS | Mobile phone data | 2500 [1500; 3800] | 350 [210; 530] | 30 |
| Pooled, raw | Mobile phone data | 2500 [1600; 3500] | 350 [220; 490] | 35 |
| Pooled, weighted | Mobile phone data | 2400 [1500; 3500] | 340 [210; 520] | 37 |
| *Facebook diffusion* | |  |  |  |
| Facebook | Facebook, with Travel survey prior | 2600 [1700; 3600] | 350 [220; 510] | 43 |
| Weighted FB | Facebook | 2400 [1600; 3300] | 330 [220; 460] | 38 |
| DHS | Facebook | 2400 [1600; 3900] | 340 [220; 540] | 32 |
| Pooled, raw | Facebook | 2400 [1500; 3500] | 330 [220; 460] | 38 |
| *Travel survey diffusion* | |  |  |  |
| Pooled, raw | Travel survey | 2500 [1800; 3400] | 340 [240; 460] | 37 |
| DHS | Travel survey | 2600 [1700; 4200] | 360 [230; 620] | 91 |
