# Supplementary material for: Comparing and integrating human mobility data sources for measles transmission modeling in Zambia
Source: PLOS Glob Public Health. 2025 May 20;5(5):e0003906. doi: 10.1371/journal.pgph.0003906 (PMC12091742; doi:10.1371/journal.pgph.0003906)
Supplement: S4 Table — (DOCX) [file pgph.0003906.s004.docx]

**S4 Table. Spearman’s rho correlation between ranks in district-level probability of introduction events, and time to introduction events, depending on the dataset used to inform mobility between districts.**

| Pairs of datasets | Probability of introduction | Time to introduction |
| --- | --- | --- |
| *Introduction in Lusaka* |  |  |
| Facebook – Mobile phone | 0.910 | 0.678 |
| Mobile phone – Travel survey | 0.758 | 0.215 |
| Facebook – Travel survey | 0.786 | 0.286 |
|  | |  |
| *Introduction in Choma and Ndola districts* | |  |
| Facebook – Mobile phone | 0.857 | 0.896 |
| Mobile phone – Travel survey | 0.831 | 0.675 |
| Facebook – Travel survey | 0.779 | 0.597 |
