# Supplementary material for: Comparing and integrating human mobility data sources for measles transmission modeling in Zambia
Source: PLOS Glob Public Health. 2025 May 20;5(5):e0003906. doi: 10.1371/journal.pgph.0003906 (PMC12091742; doi:10.1371/journal.pgph.0003906)
Supplement: S1 Text — Availability of data on diffusion, fitting an exponential gravity model, assessing goodness of fit. (DOCX) [file pgph.0003906.s005.docx]

**S1 Text. Modeling diffusion processes**

Availability of data on diffusion

Information on diffusion in Facebook data and travel survey was sparse, with only 19 and 39 destinations recorded as having non-zero number of trips. In comparison, mobile phone data provided non-zero number of trips into 115 districts, for a total of 11,342 origin-destination pairs, and included a wider range in reported trip counts (see S4 Fig). From Ndola district, there was travel to two other districts in Facebook data, to 26 districts in travel survey, and 99 districts in mobile phone data (see S6 Fig).

Fitting an exponential gravity model

In the exponential gravity model, which best fit data from all three sources (see below for goodness of fit metrics), the number of trips from district $i$ to district $j$, $T_{ij}$, is defined by

$$T_{ij}\sim Poisson(\mu_{ij})$$

$$\mu_{ij}=\frac{\theta N_{i}^{\omega_{i}}N_{j}^{\omega_{j}}}{e^{d_{ij}/\delta}}$$

Where $T_{ij}$ is the number of trips from district $i$ to district $j$, with $i$ and $j$ distinct; $N_{i}$ is the population size of the origin district; $N_{j}$ is the population size of the destination district; and $d_{ij}$ is the distance between the centroids of the two districts, in kilometers. The $\omega_{i}$ and $\omega_{j}$ parameters modify the contribution of origin and destination population sizes, respectively, while $\delta$ is the distance deterrent parameter, modifying the extent to which distance between origin and destination reduces the probability of travel.

Formulations of the other tested models are provided elsewhere [1]*.*

Assessing goodness of fit

After fitting different gravity models (basic, transport, power law, exponential, normalized power law, normalized exponential, and scaled power law), we assessed model fit using the goodness of fit metrics (deviance information criterion (DIC), root-mean-square error (RMSE), mean absolute percentage error (MAPE), and R^2^ and summary plots for the fitted model and selected a best-fit model for each dataset. Key summaries of these metrics are presented below.

**References**

1. Giles JR, Wesolowski A. A curated list of mobility models. [cited 24 Oct 2022]. Available: https://covid-19-mobility-data-network.github.io/mobility/articles/V5_list_models.html

## Summary of goodness of fit metrics for gravity models fit to different mobility datasets

Mobile phone data


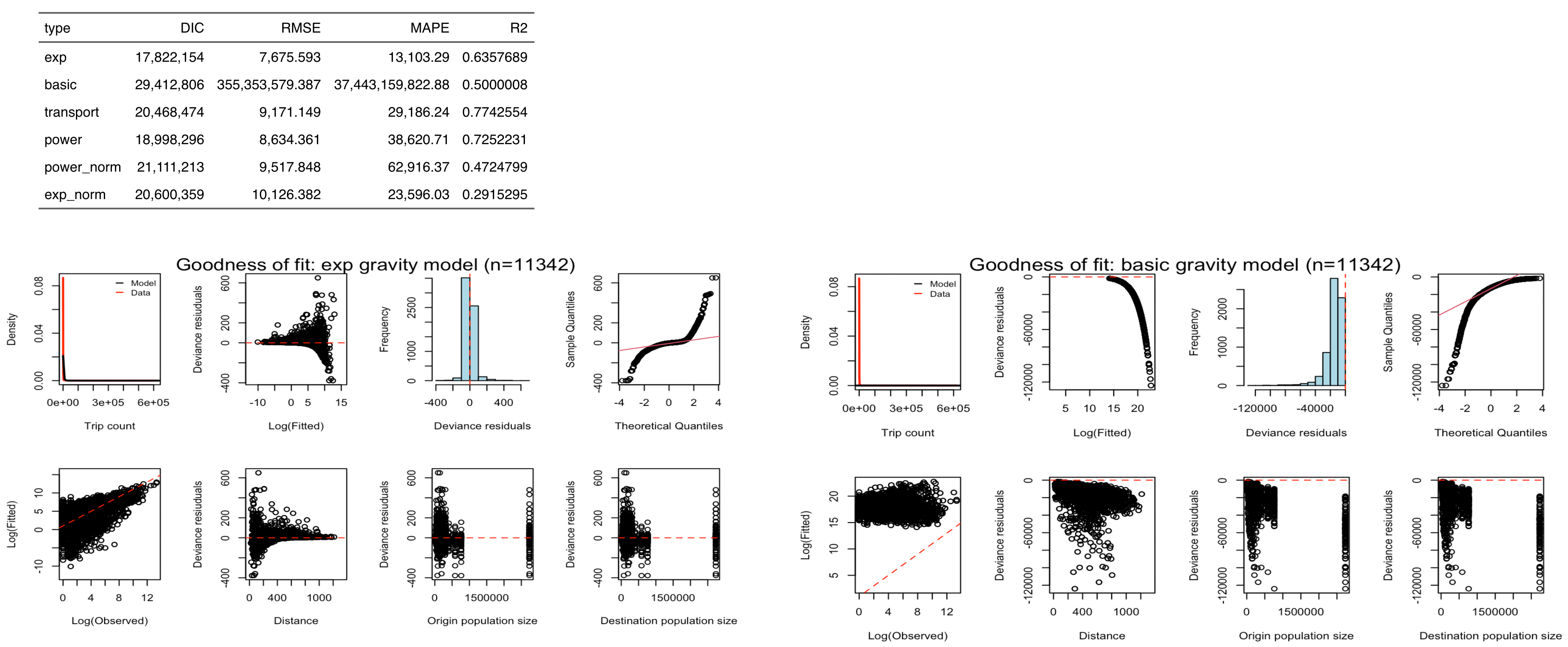


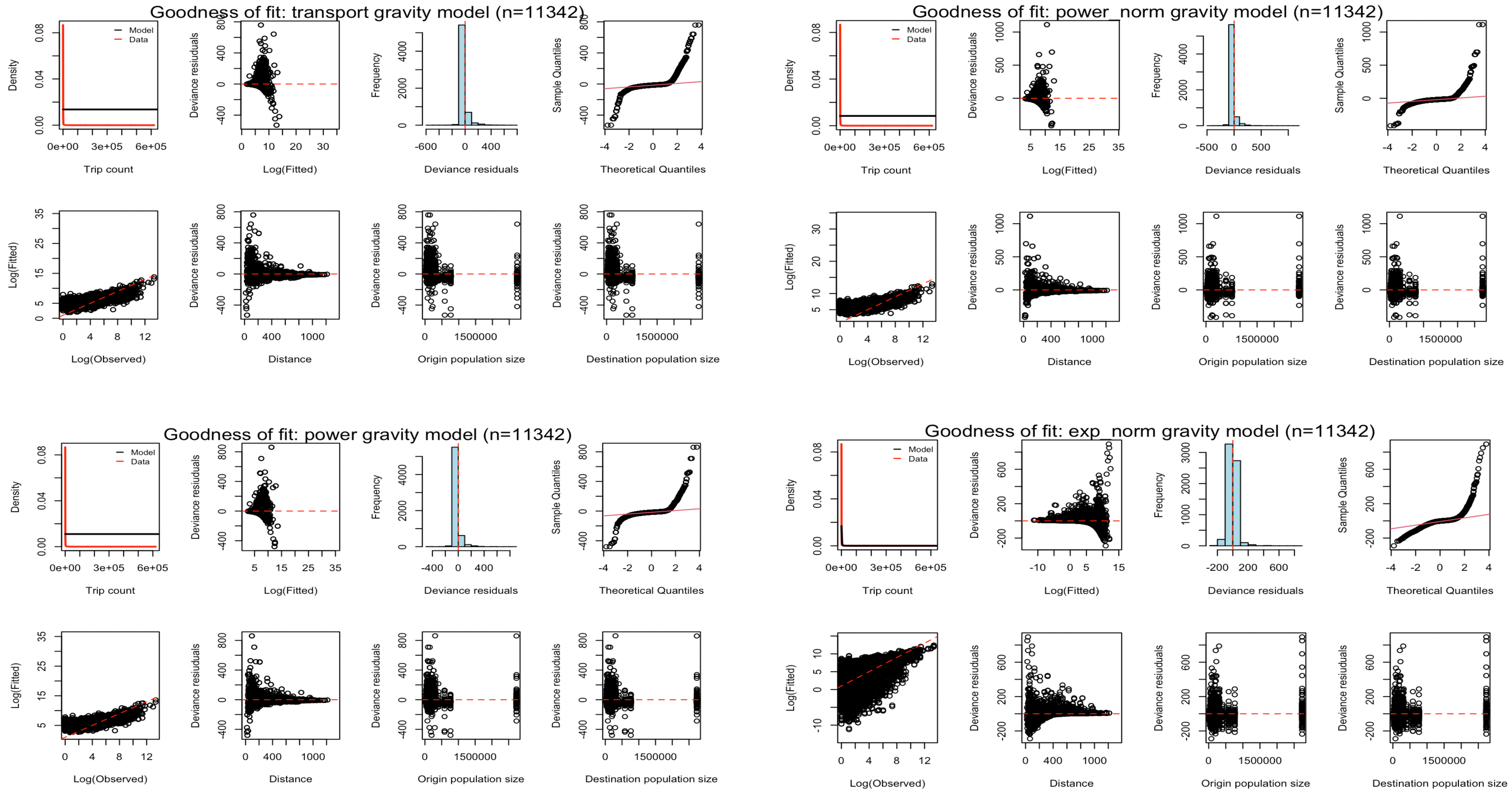


Facebook data


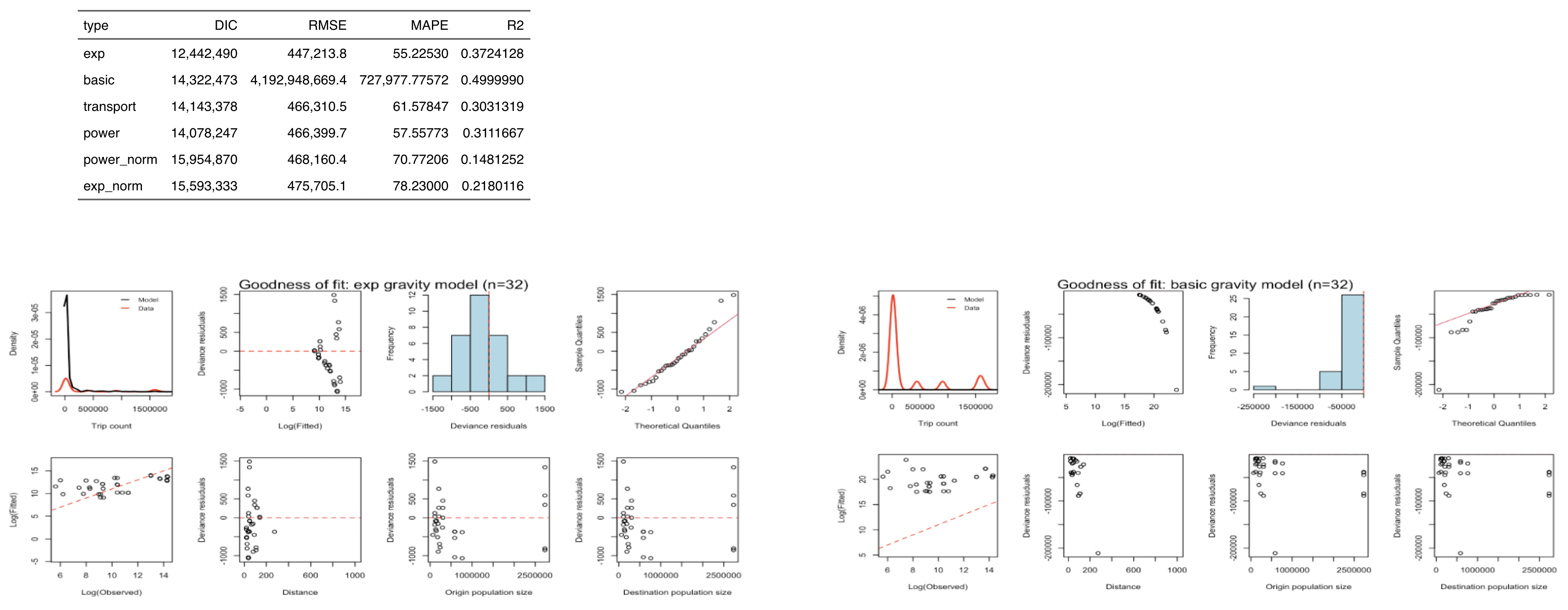


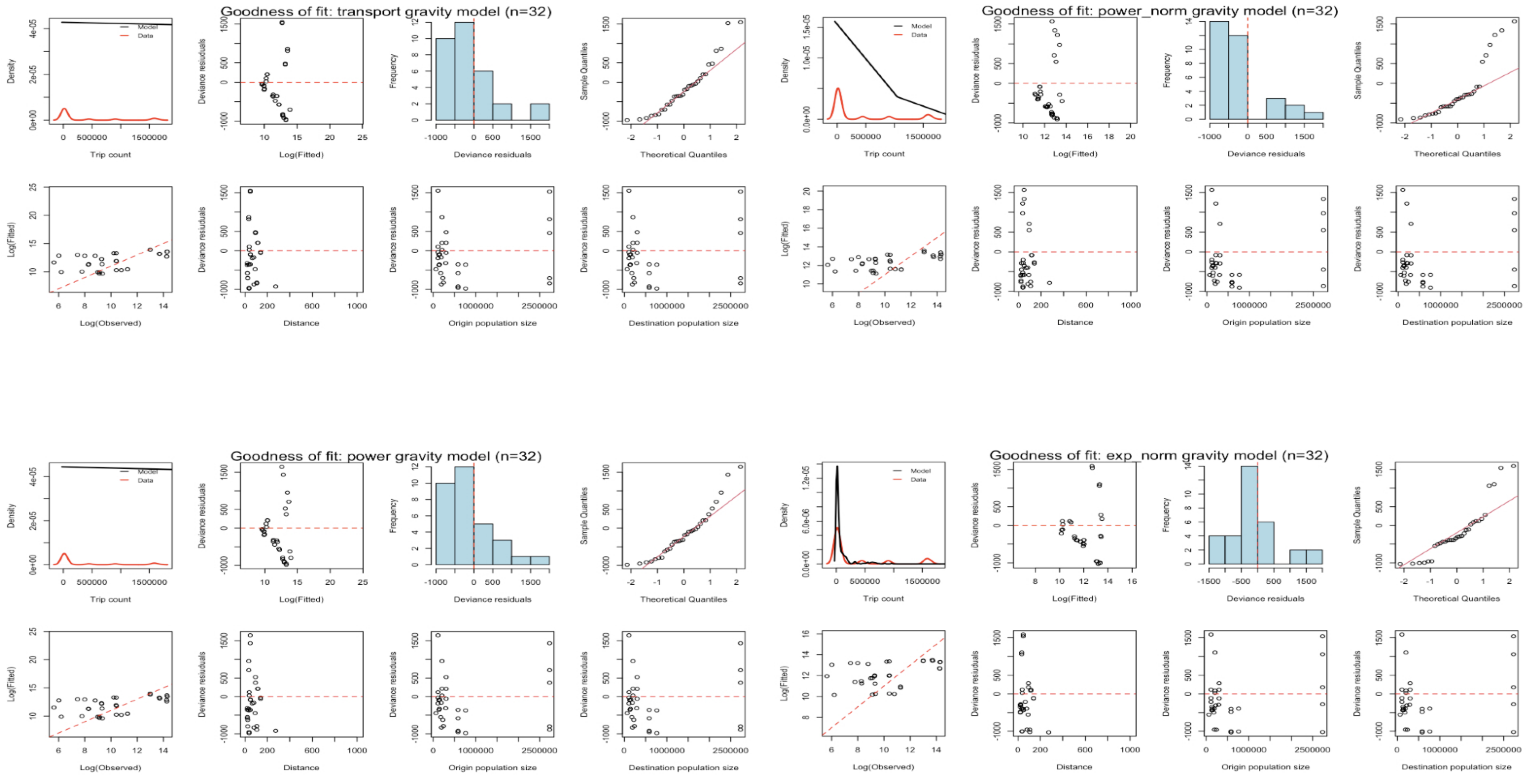


Travel survey


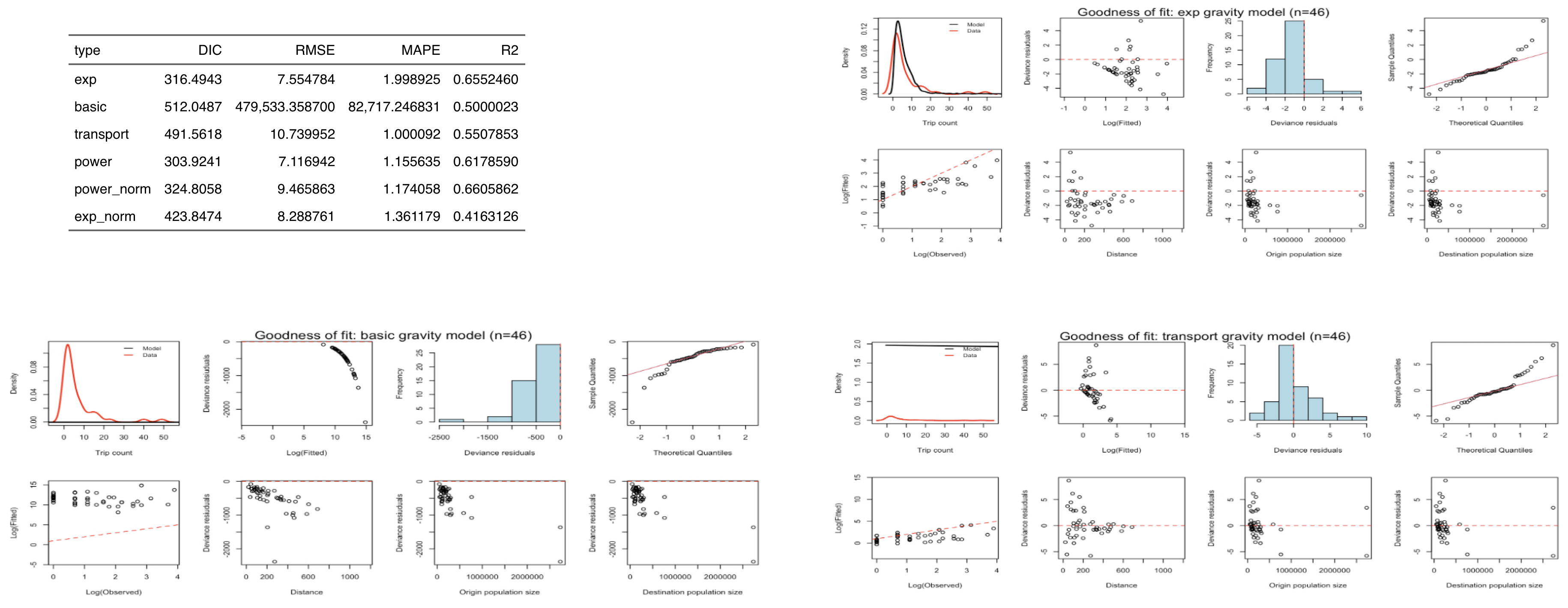


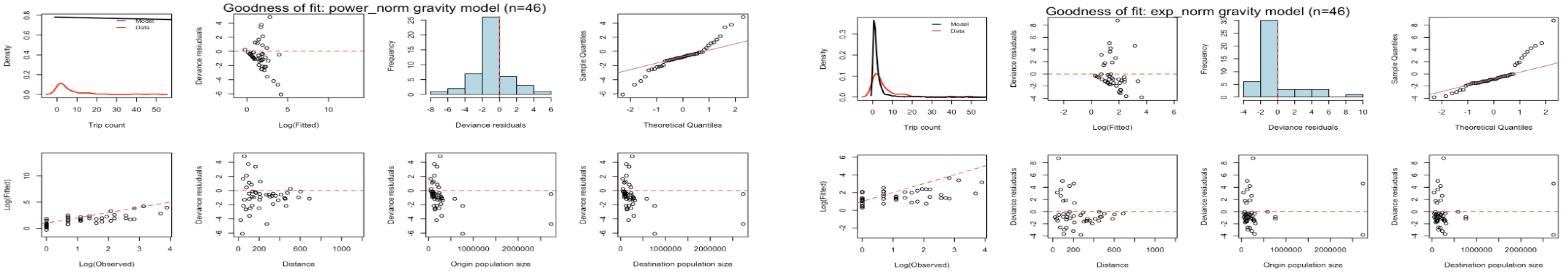


Comparing coefficients

The overall best-fitting model was based on the travel survey data. This may be due to the limited geographic coverage of the travel survey, with only 39 destinations included in the analysis, along with two origin districts where the travel survey was carried out. As a result, the travel survey did not include origin-destination pairs that didn’t report travel, and likely excluded low-travel routes from the two districts of origin. This was in high contrast with fitting mobile phone data; the latter did not perform well when fitting low-travel routes (those origin-destination pairs with very low probability of travel), but had a very high total sample size, resulting in highly precise coefficient estimates.

For the best-fitting gravity model, per data set, when we compared estimated coefficients, we found that estimates were consistent between the mobile phone and Facebook data sets. In contrast, the travel survey distance parameter was nearly four times higher than either the mobile phone- or Facebook-derived parameter (see Fig 3), suggesting that the trip distances in this survey displayed a smaller deterrent of travel. The origin population size coefficient also differed in the travel survey, with almost no importance placed on this variable when estimating flows (see Fig 3). There was substantial variability in the coefficient for the destination population size (between 0.7-1.1), suggesting that larger destination population size had a bigger role in attracting trips in the diffusion process estimated from Facebook data compared to the mobile phone and travel survey data. In general, precise estimates were only obtainable for the model fitted to mobile phone data and, to a lesser degree, Facebook data (see Fig 3).
