# Supplementary material for: Comparing and integrating human mobility data sources for measles transmission modeling in Zambia
Source: PLOS Glob Public Health. 2025 May 20;5(5):e0003906. doi: 10.1371/journal.pgph.0003906 (PMC12091742; doi:10.1371/journal.pgph.0003906)
Supplement: S2 Text — (DOCX) [file pgph.0003906.s006.docx]

**S2 Text. Incorporating information from priors and observed data using Bayesian framework**

The Bayes’ rule is formulated as

$p\left( \theta| y \right)=\frac{p(y|\theta)p(\theta)}{\int_{\Theta} p\left( y | \tilde{\theta} \right)p\left( \tilde{\theta} \right)d\tilde{\theta}}$,

Where $p(\theta)$ is the prior distribution for parameter $\theta$ with parameter space $\Theta$, $y$ is the observed dataset, and $p(y|\theta)$ is the sampling model, describing our belief that $y$ would be the outcome of our study if we knew $\theta$ to be true [1].

As an illustrative example, let’s suppose we observe data, whose likelihood is drawn from a normal distribution with mean = 60 and standard deviation = 5 (n=100). Our prior distribution is drawn from a truncated normal distribution with mean = 50 and standard deviation = 6 (n=10,000). The prior is informative, and the posterior distribution can be observed in the figure below, borrowing information from both the prior and the likelihood.


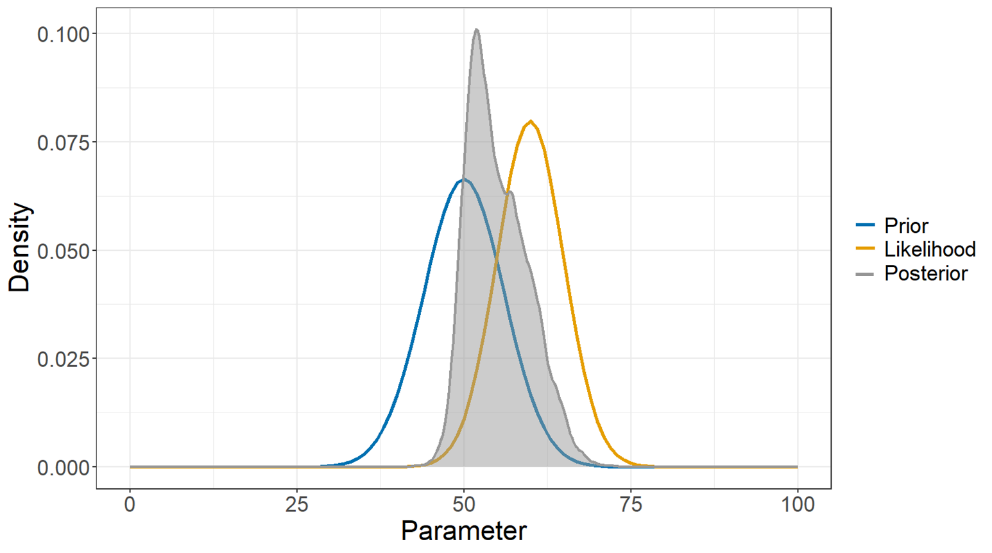


**Reference**

1. Hoff PD. A First Course in Bayesian Statistical Methods. New York, NY: Springer; 2009. doi:10.1007/978-0-387-92407-6
