# Supplementary material for: Comparing and integrating human mobility data sources for measles transmission modeling in Zambia
Source: PLOS Glob Public Health. 2025 May 20;5(5):e0003906. doi: 10.1371/journal.pgph.0003906 (PMC12091742; doi:10.1371/journal.pgph.0003906)
Supplement: S3 Text — (DOCX) [file pgph.0003906.s007.docx]

## **S3 Text. Transmission model specification**

To model measles transmission, we used a discrete-time spatial compartmental model, with parameters and initial conditions informed by data from Zambia.

We used 115 districts of Zambia as the spatial units in the model. The shapefile with the districts is openly available (<https://data.humdata.org/dataset/cod-ab-zmb>?). Time steps were set to measles generation time.

At beginning of each time step, individuals moved across districts based on probabilities defined in a mobility matrix, wherein the probability of departure for each district was estimated from the fitted departure model, and the probability of travel from district $i$ to $j$, where $i\neq j$, predicted by the fitted diffusion model.

After moving individuals across spatial units, we used a multinomial draw to move individuals across compartments (either Maternal Immunity, Susceptible, Infected / infectious, Vaccinated, and Removed).

We assumed that vaccination can be administrated through either routine or campaign approach. Vaccine was set to be imperfect; vaccinated individuals either moved to recovered compartment, or did not develop immunity and remained susceptible to infection. We considered receipt of two doses of measles-containing vaccine to be immunizing.

We used the following set of parameters in the model:

Rate of waning immunity = 0.45

Birth rate: province-level birth rate, obtained from administrative records

Background rate of death: province-level crude mortality rate, divided by 26, obtained from administrative records

Rate of death from measles = 0.0013 [1]

Mixing parameter = 0.975 [2]

Recovery rate = 1 (because time between time steps was set to generation time)

Effectiveness of first dose of measles-containing vaccine administered through routine approach = 93%

Rate of receipt of first dose of vaccine through routine approach = (9 months – 1 / rate of waning immunity) ~ about 13 weeks

Rate of receipt of second dose of vaccine through routine approach = 9 months (average time between eligibility for first and second dose of MCV)

Population size in each district was obtained from the district-level projections, based on the 2010 Zambia census [3]. We assumed that 5% of children younger than 5 years were immune (in the Maternal Immunity compartment), roughly corresponding to the number of children under 3 months.

We used the Zambia Health Management Information System to obtain district-level vaccination coverage for 2018. To estimate vaccination coverage for years after 2018, we scaled the district-level estimates using nationwide estimates of MCV1 and MCV2 coverage [4]. For coverage estimates larger than 99%, we set them to 99%. We incorporated supplementary immunization activities for 2016 and 2020, with coverage estimates informed by Post-Coverage Evaluation Survey reports [5,6]. As the coverage estimates were province-level, we assumed that districts within each province had the same levels of coverage.

Initial proportion of population susceptible to measles was determined by estimates from the 2016 national serological survey [7]. For each simulation, we drew district-level proportion immune from a random distribution with bounds specified in the serological survey.

**References**

1. Portnoy A, Jit M, Ferrari M, Hanson M, Brenzel L, Verguet S. Estimates of case-fatality ratios of measles in low-income and middle-income countries: a systematic review and modelling analysis. The Lancet Global Health. 2019;7: e472–e481. doi:10.1016/S2214-109X(18)30537-0

2. Glass K, Xia Y, Grenfell BT. Interpreting time-series analyses for continuous-time biological models--measles as a case study. J Theor Biol. 2003;223: 19–25. doi:10.1016/s0022-5193(03)00031-6

3. Central Statistical Office. Population and Demographic Projections, 2011-2035 - Zambia Data Portal. Jul 2013 [cited 24 Oct 2022]. Available: https://zambia.opendataforafrica.org/ZMPHC2015/population-and-demographic-projections-2011-2035#

4. WHO, UNICEF. Zambia: WHO and UNICEF estimates of national immunization coverage: 2021 revision. UNICEF Data; 2022 Jul. Available: https://data.unicef.org/resources/dataset/immunization/

5. Ministry of Health Zambia. Post Coverage Measles-Rubella Campaign Evaluation Survey. Government of the Republic of Zambia; 2021 Nov.

6. Ministry of Health Zambia. Post MR Campaign Survey Report. Government of the Republic of Zambia; 2017 Mar.

7. Carcelen AC, Winter AK, Moss WJ, Chilumba I, Mutale I, Chongwe G, et al. Leveraging a national biorepository in Zambia to assess measles and rubella immunity gaps across age and space. Sci Rep. 2022;12: 10217. doi:10.1038/s41598-022-14493-3
