# Supplementary material for: Comparing and integrating human mobility data sources for measles transmission modeling in Zambia
Source: PLOS Glob Public Health. 2025 May 20;5(5):e0003906. doi: 10.1371/journal.pgph.0003906 (PMC12091742; doi:10.1371/journal.pgph.0003906)

## **S1 Fig. Origin-destination matrices for three datasets with information on diffusion processes in Zambia. A. Mobile phone data, B. Facebook dataset, C. Travel survey.**

**
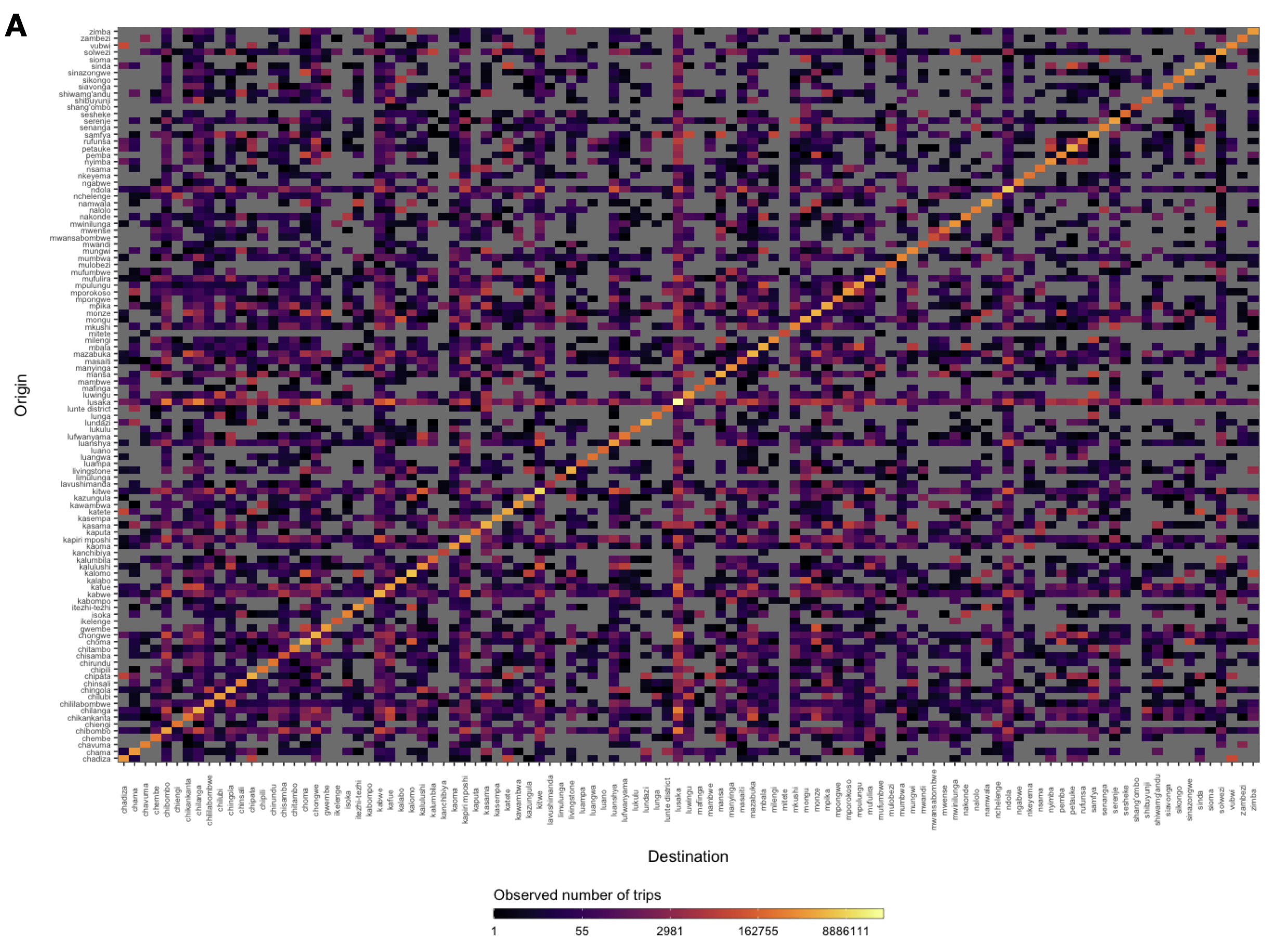
**

**
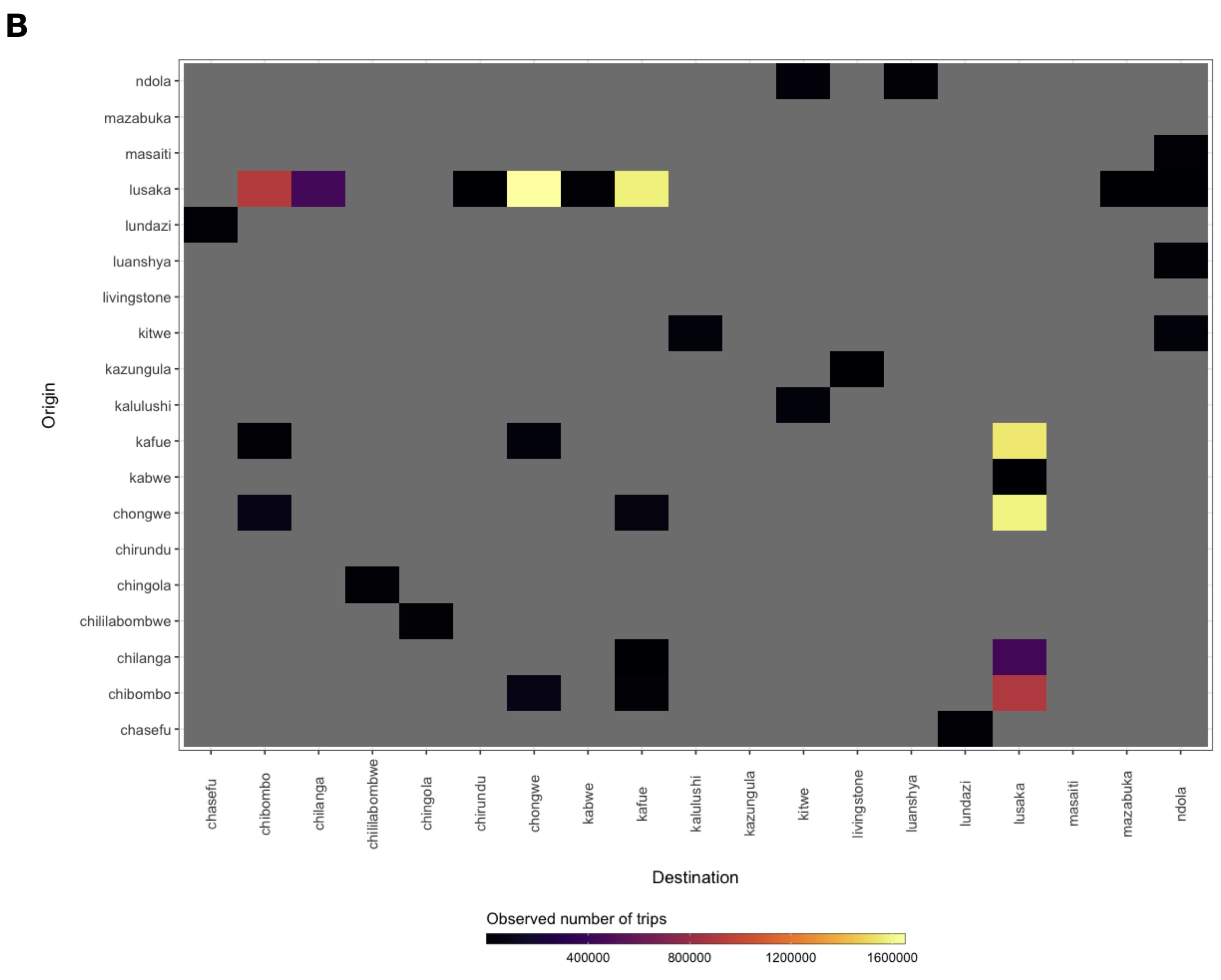
**


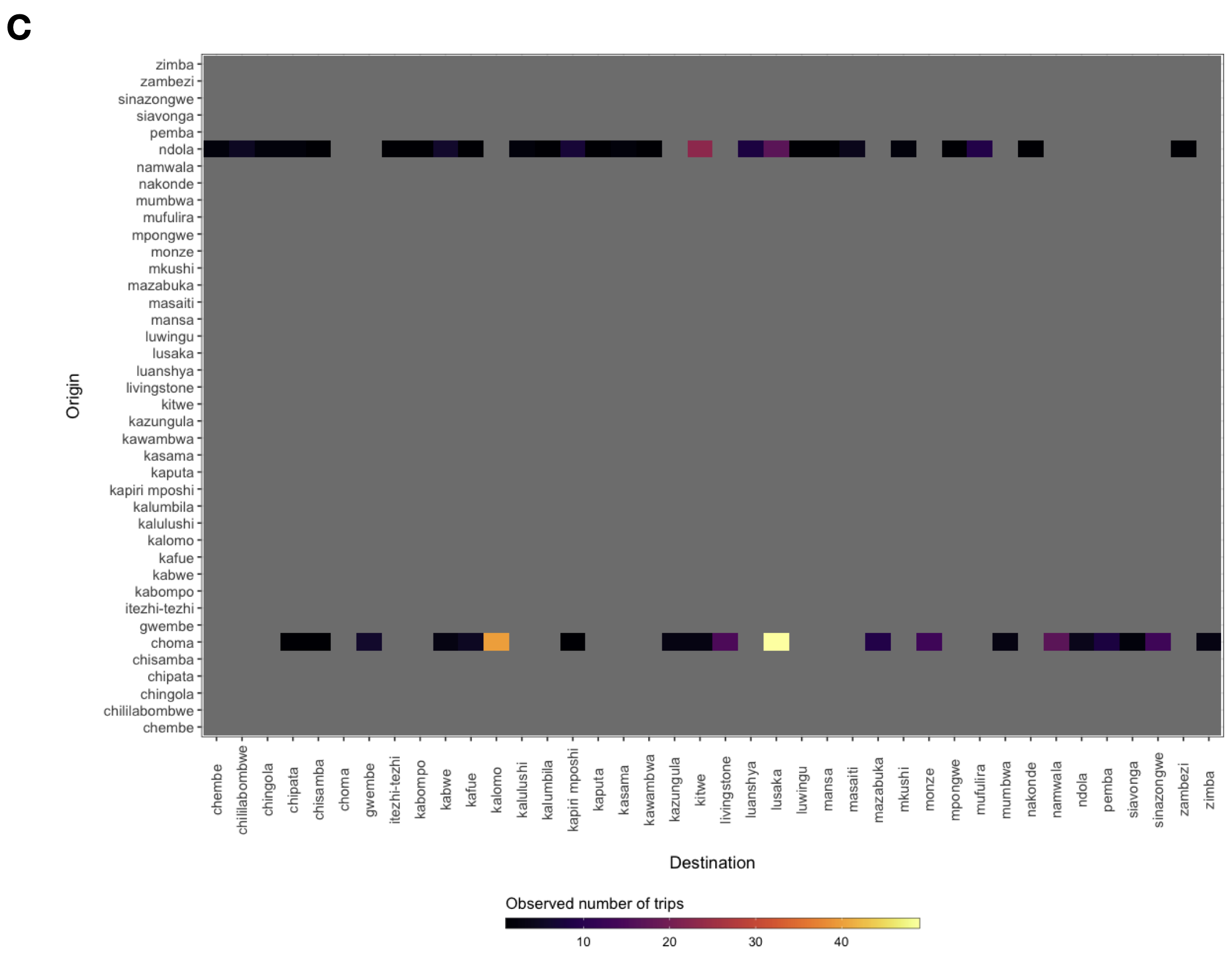

Supplement: S1 Fig — A. Mobile phone data, B. Facebook dataset, C. Travel survey. (DOCX) [file pgph.0003906.s008.docx]
