# Supplementary material for: Comparing and integrating human mobility data sources for measles transmission modeling in Zambia
Source: PLOS Glob Public Health. 2025 May 20;5(5):e0003906. doi: 10.1371/journal.pgph.0003906 (PMC12091742; doi:10.1371/journal.pgph.0003906)

## **S2 Fig. Observed probabilities of departure from district from Mobile phone dataset, Facebook, Demographic and Health Survey (DHS), and Travel survey.**

## A. Boxplots of district-level probabilities of departure, by province. Outliers have been removed from the plot to facilitate visibility. The travel survey was only done in two districts, in Copperbelt and Southern provinces, respectively; no data are available from the survey in other districts and provinces. Facebook data were only available for districts in four provinces. B. The probability of travel by district, in selected districts. Choma and Ndola were included as the only districts with Travel survey data. Other districts are the five districts with the largest difference in probability of departure across datasets. Observed probabilities of departure from district from Mobile phone dataset, Facebook, Demographic and Health Survey (DHS), and Travel survey. C. All observed probabilities of departures for the four datasets.


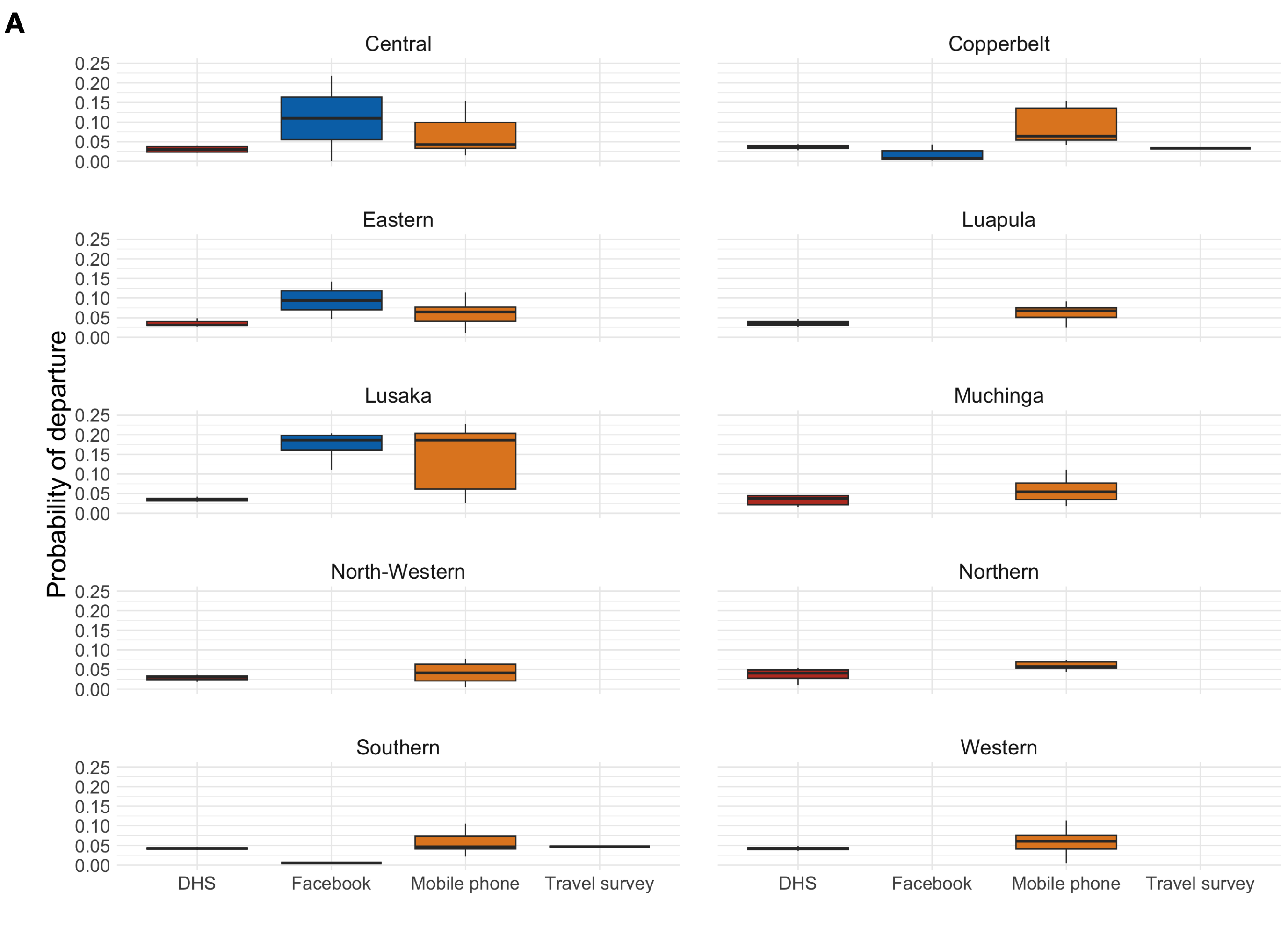


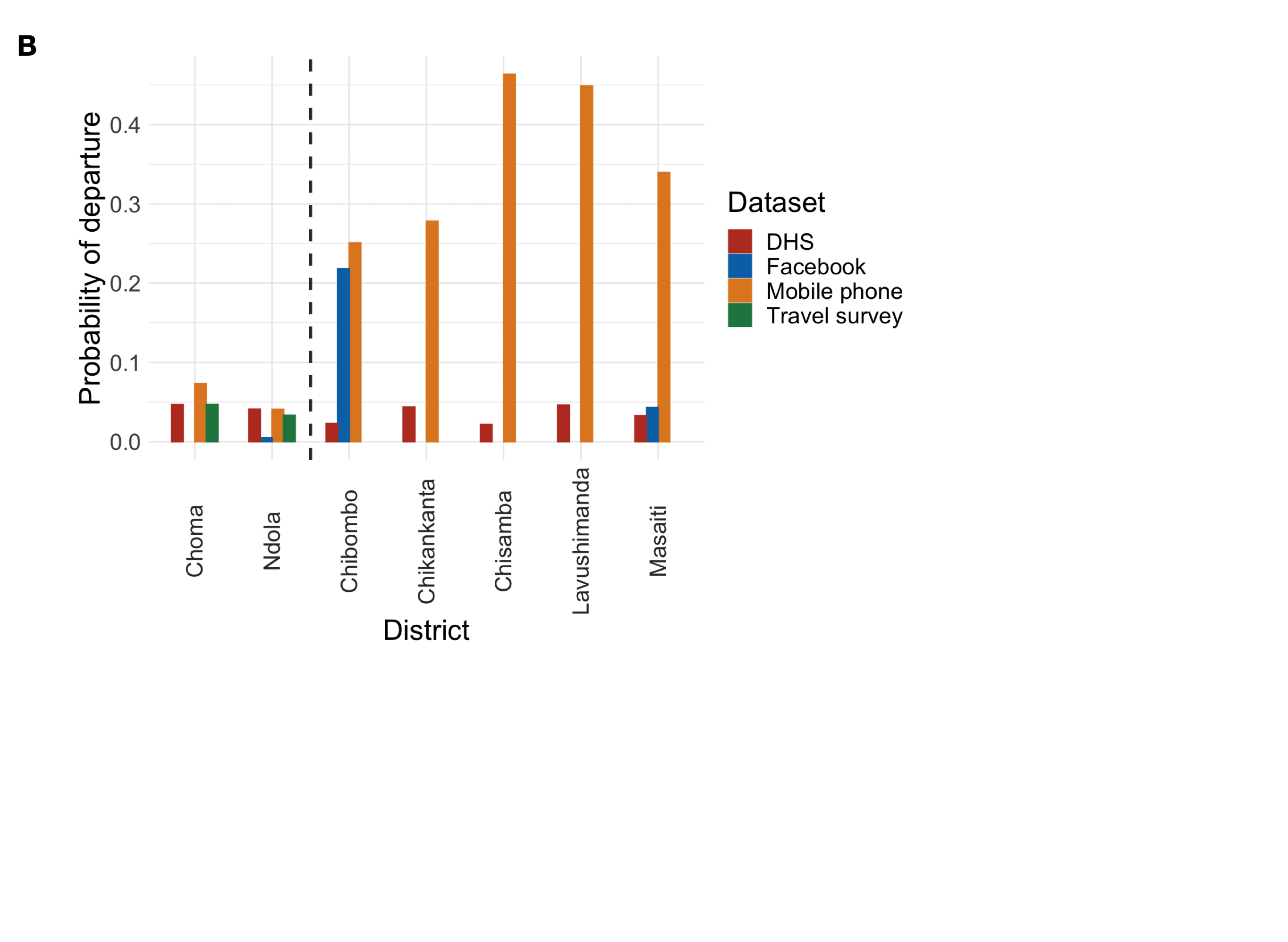


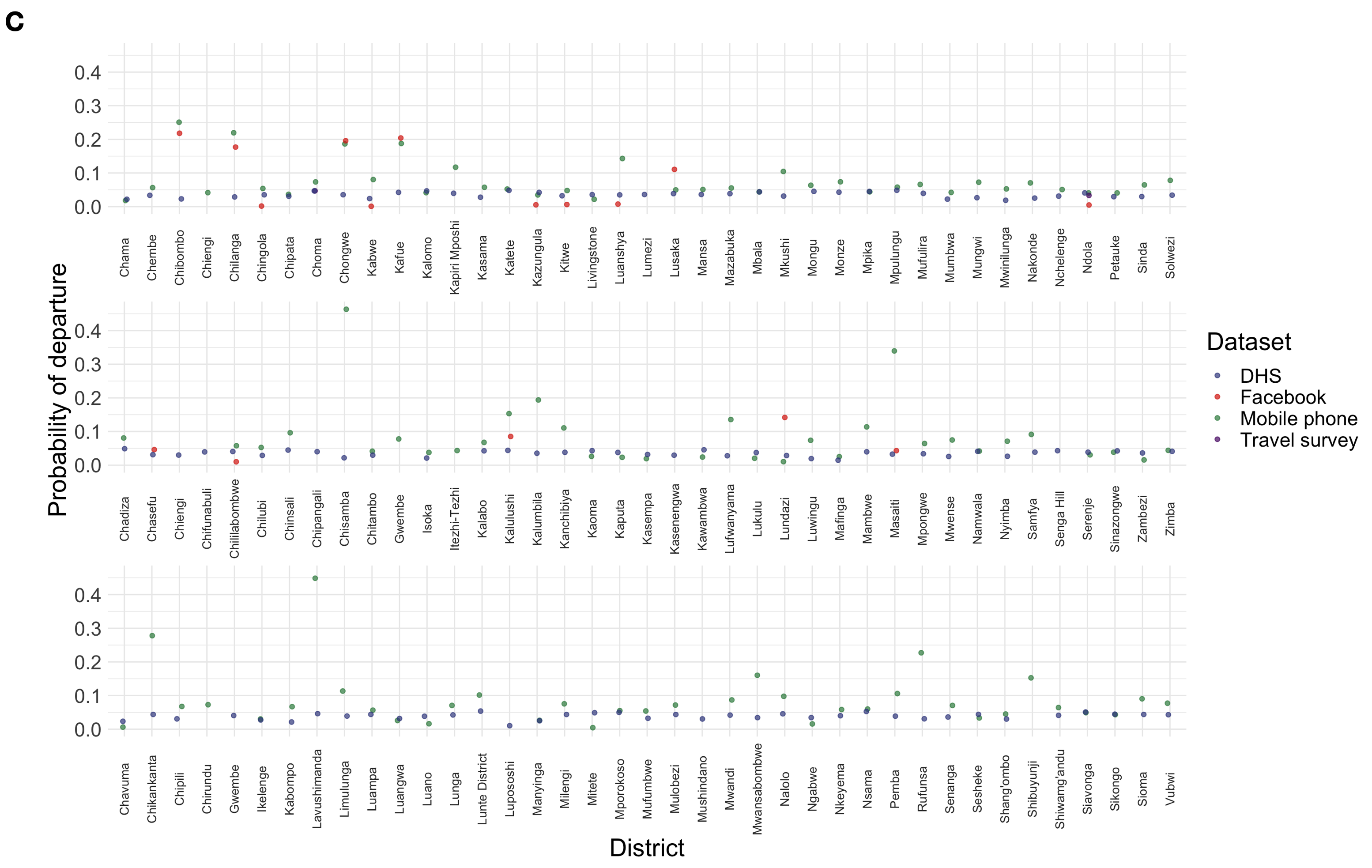

Supplement: S2 Fig — A. Boxplots of district-level probabilities of departure, by province. Outliers have been removed from the plot to facilitate visibility. The travel survey was only done in two districts, in Copperbelt and Southern provinces, respectively; no data are available from the survey in other districts and provinces. Facebook data were only available for districts in four provinces. B. The probability of travel by district, in selected districts. Choma and Ndola were included as the only districts with Travel survey data. Other districts are the five districts with the largest difference in probability of departure across datasets. Observed probabilities of departure from district from Mobile phone dataset, Facebook, Demographic and Health Survey (DHS), and Travel survey. C. All observed probabilities of departures for the four datasets. (DOCX) [file pgph.0003906.s009.docx]
