# Supplementary material for: Comparing and integrating human mobility data sources for measles transmission modeling in Zambia
Source: PLOS Glob Public Health. 2025 May 20;5(5):e0003906. doi: 10.1371/journal.pgph.0003906 (PMC12091742; doi:10.1371/journal.pgph.0003906)

S3 Fig. Probability of travel out of the district, comparing estimates from the beta-binomial model fit to raw datasets, and pooled estimates.

A. Probability of travel, where pooled value was obtained by pooling estimates from raw Mobile phone data, Demographic and Health Survey (DHS), Facebook, and Travel survey. B. Probability of travel, where pooled values were obtained by pooling estimates from DHS and Travel survey, and weighted Mobile phone and Facebook data. C. Comparison of probabilities of travel using pooled estimates from raw datasets (Mobile phone data, Facebook, Travel survey, and Demographic and Health survey) and the mixture of raw and weighted datasets (weighted Mobile phone data, weighted Facebook, Travel survey, and Demographic and Health survey). Each point represents a district. X-axis is the probability of leaving from pooled estimates from values obtained through fitting the beta-binomial model to raw datasets. The diagonal line indicates a boundary of no change in probabilities.

**
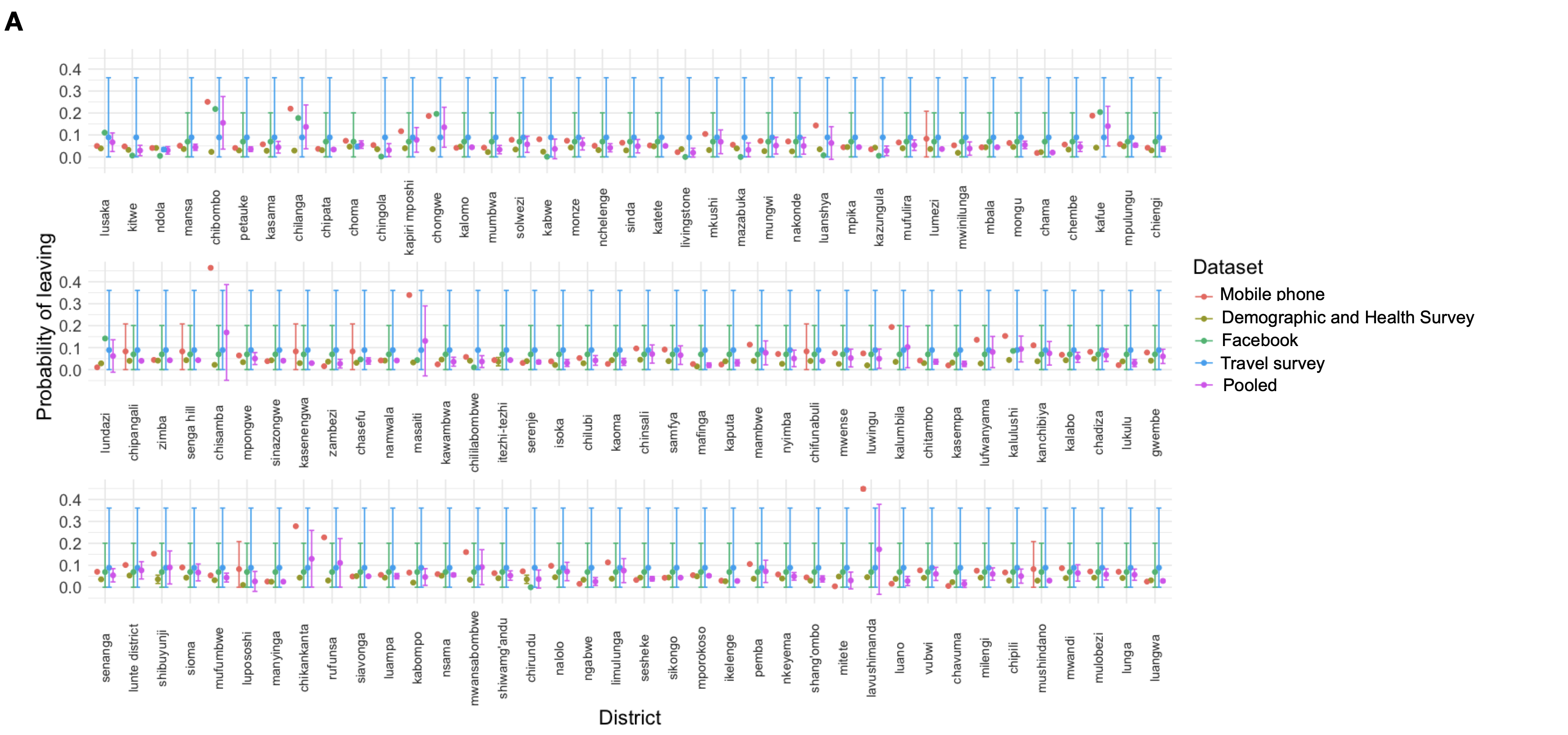
**


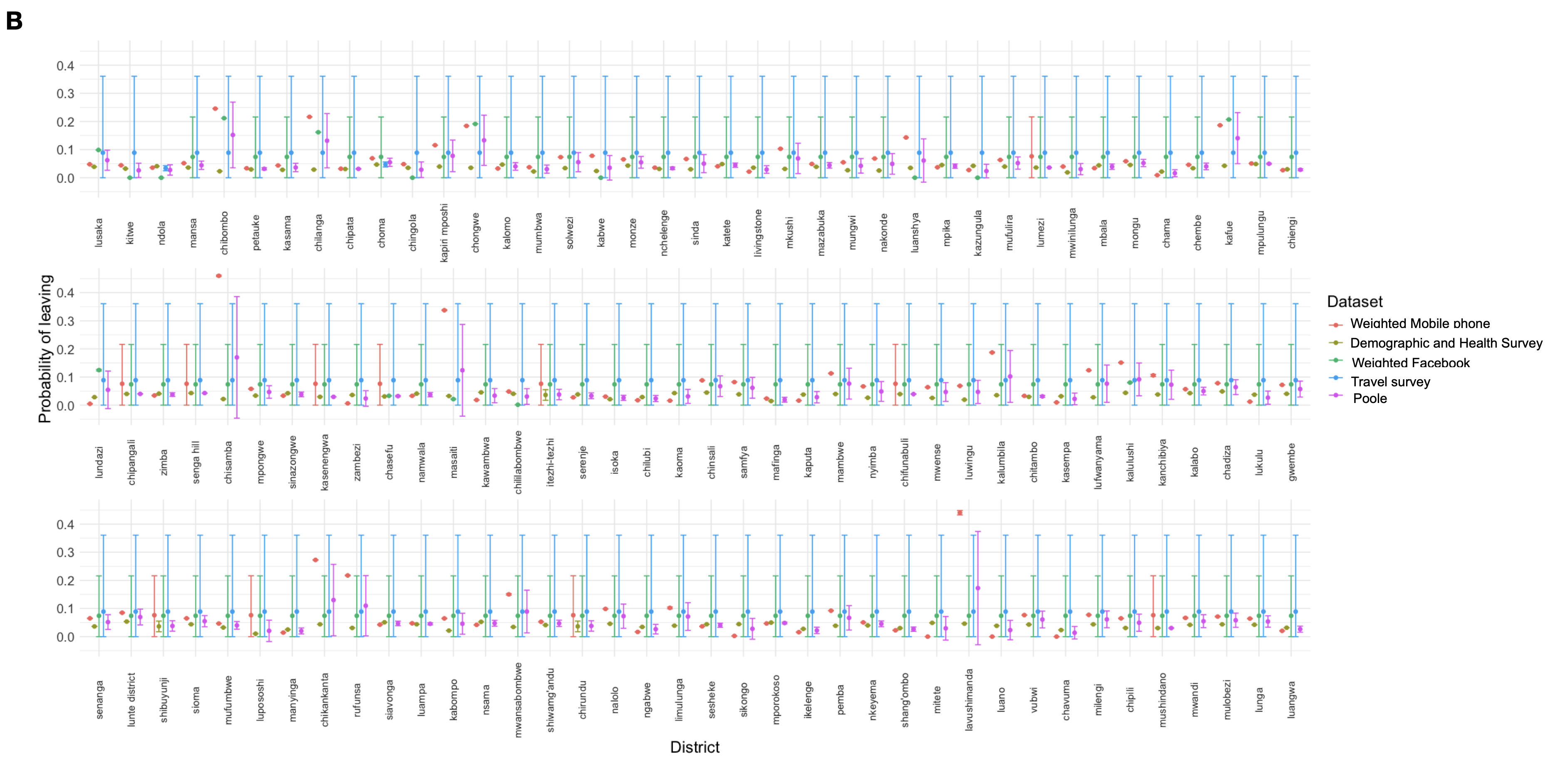


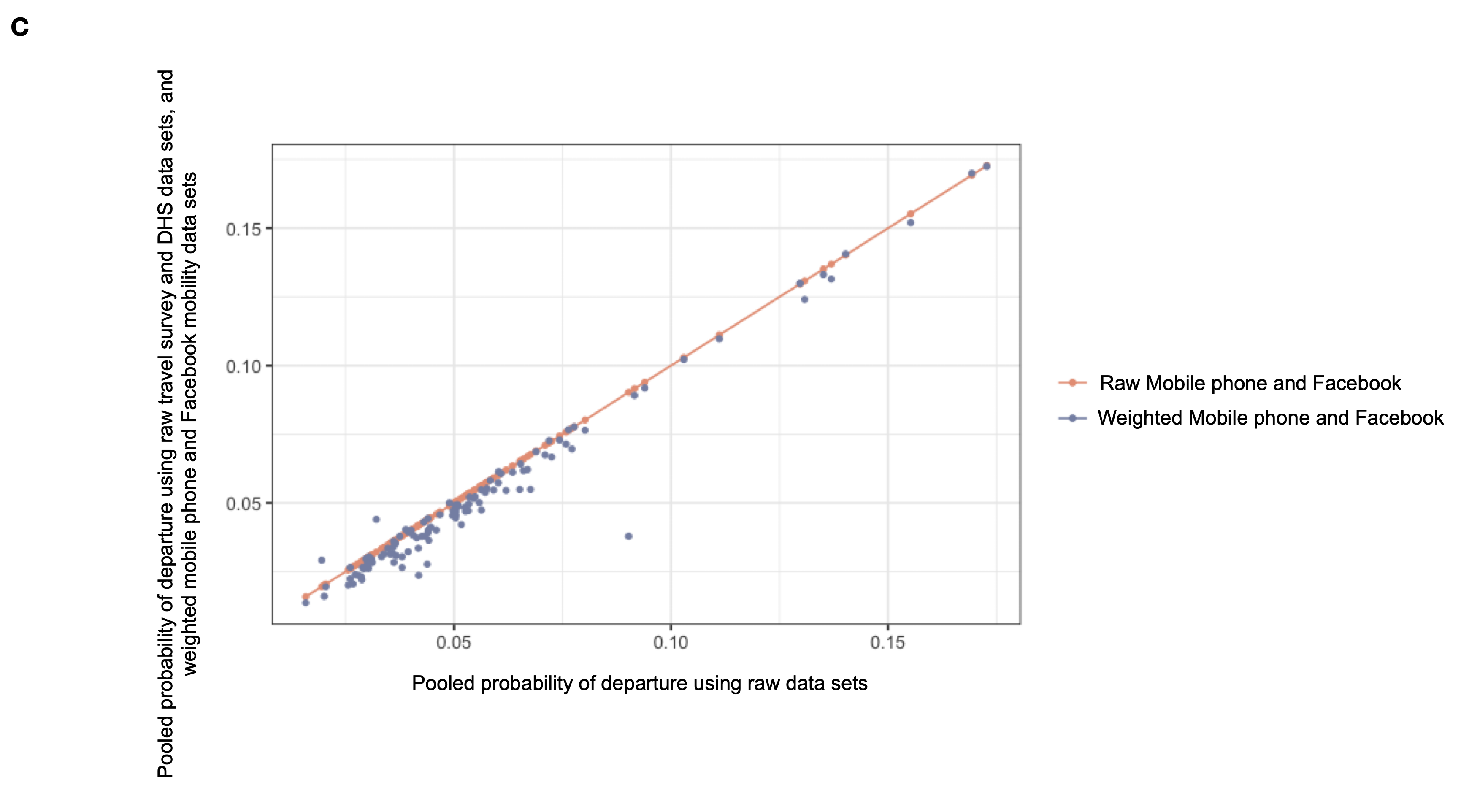

Supplement: S3 Fig — A. Probability of travel, where pooled value was obtained by pooling estimates from raw Mobile phone data, Demographic and Health Survey (DHS), Facebook, and Travel survey. B. Probability of travel, where pooled values were obtained by pooling estimates from DHS and Travel survey, and weighted Mobile phone and Facebook data. C. Comparison of probabilities of travel using pooled estimates from raw datasets (Mobile phone data, Facebook, Travel survey, and Demographic and Health survey) and the mixture of raw and weighted datasets (weighted Mobile phone data, weighted Facebook, Travel survey, and Demographic and Health survey). Each point represents a district. X-axis is the probability of leaving from pooled estimates from values obtained through fitting the beta-binomial model to raw datasets. The diagonal line indicates a boundary of no change in probabilities. (DOCX) [file pgph.0003906.s010.docx]
