# Supplementary material for: Comparing and integrating human mobility data sources for measles transmission modeling in Zambia
Source: PLOS Glob Public Health. 2025 May 20;5(5):e0003906. doi: 10.1371/journal.pgph.0003906 (PMC12091742; doi:10.1371/journal.pgph.0003906)

**S4 Fig. Proportion of districts in Zambia with measles introduction events, as observed in simulations of measles transmission dynamics with population composed of a sub-population whose movement patterns are captured by mobile phone data, and sub-population whose movement patterns are captured by travel survey.**


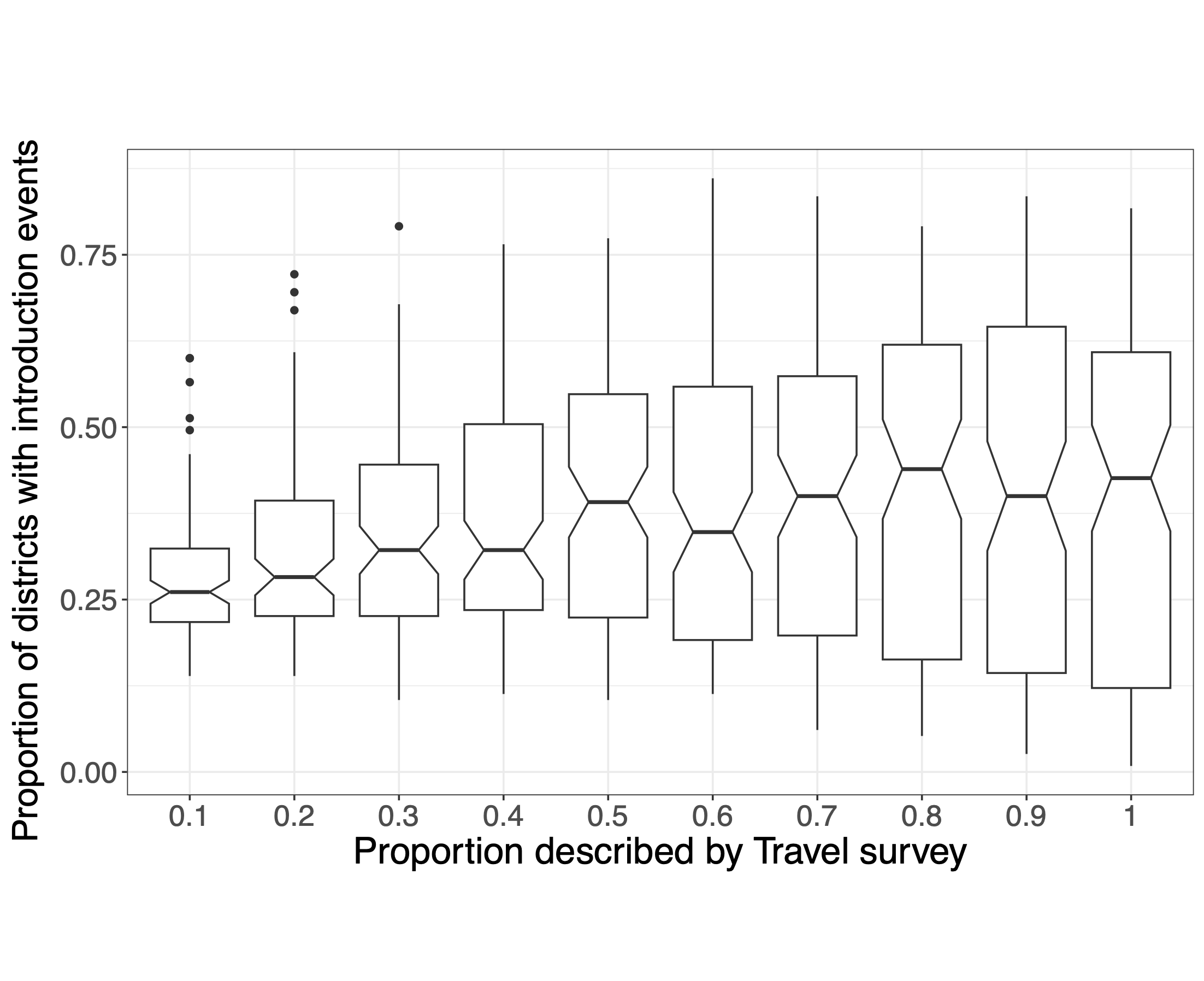

Supplement: S4 Fig — (DOCX) [file pgph.0003906.s011.docx]
