# Supplementary material for: Comparing and integrating human mobility data sources for measles transmission modeling in Zambia
Source: PLOS Glob Public Health. 2025 May 20;5(5):e0003906. doi: 10.1371/journal.pgph.0003906 (PMC12091742; doi:10.1371/journal.pgph.0003906)

S5 Fig. Results of sensitivity analysis: simulations of measles dynamics following the introduction of 5 cases into Ndola district and 5 cases into Choma district. A. Cumulative measles cases in scenarios where a single data set was used to inform departure and diffusion processes. B. Proportion of districts with introduction events, with mobility between districts informed by different combinations of datasets. C. Proportion of districts with introductions, with panels representing dataset used to inform diffusion process. D. Cumulative cases, with panels representing dataset used to inform diffusion process. E. Mean cumulative cases of measles from simulation, with results split by which dataset was used to inform diffusion process.


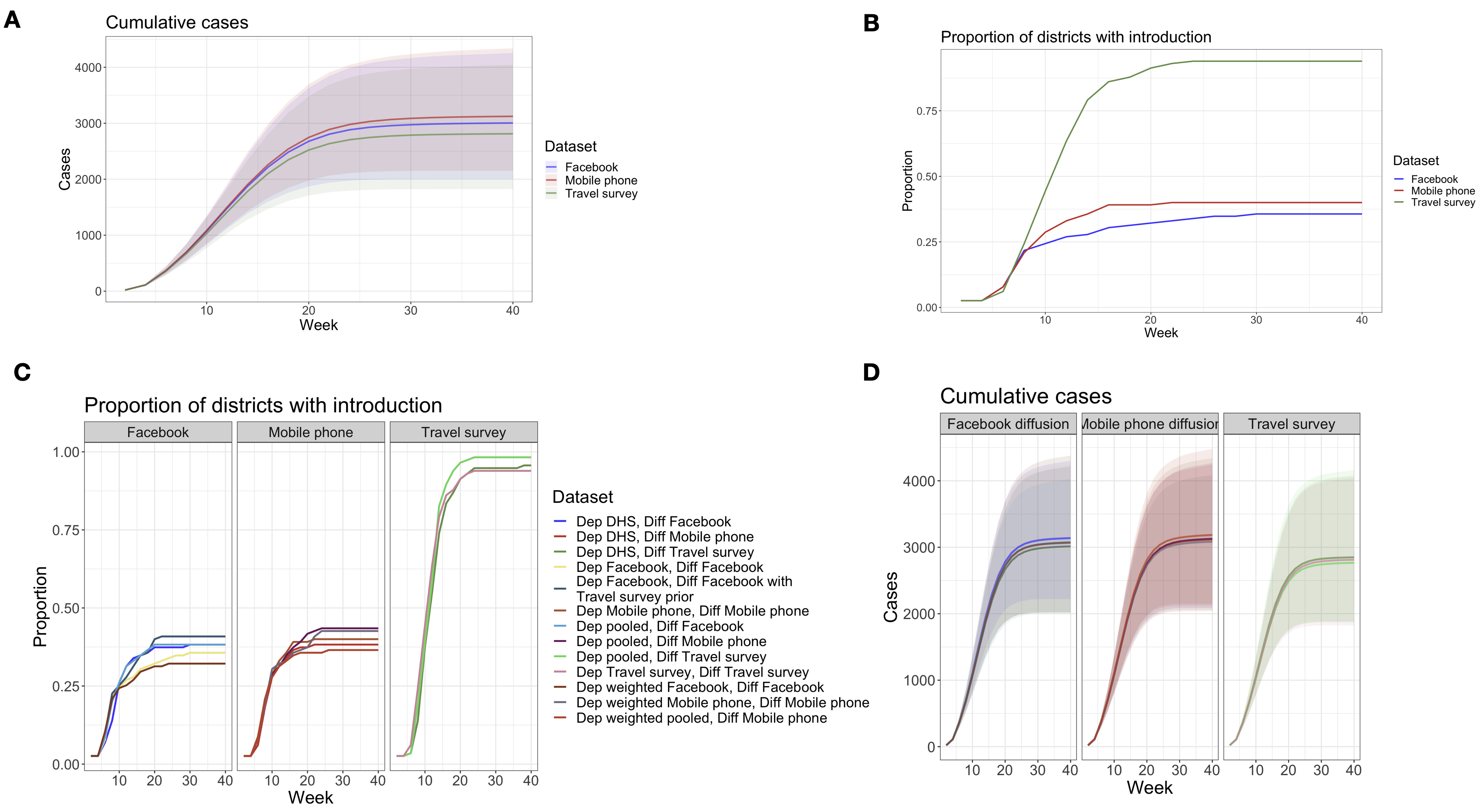


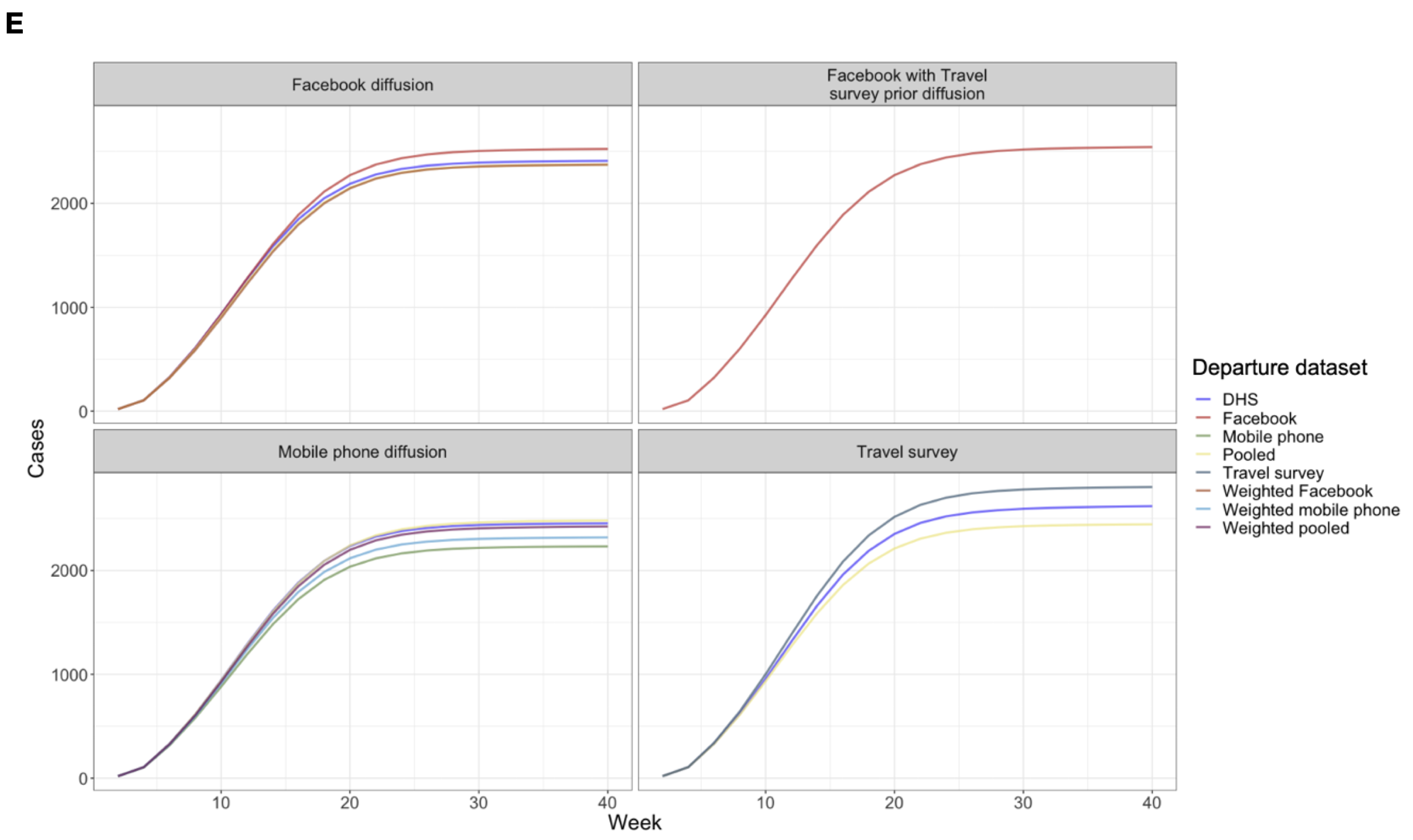

Supplement: S5 Fig — A. Cumulative measles cases in scenarios where a single data set was used to inform departure and diffusion processes. B. Proportion of districts with introduction events, with mobility between districts informed by different combinations of datasets. C. Proportion of districts with introductions, with panels representing dataset used to inform diffusion process. D. Cumulative cases, with panels representing dataset used to inform diffusion process. E. Mean cumulative cases of measles from simulation, with results split by which dataset was used to inform diffusion process. (DOCX) [file pgph.0003906.s012.docx]
