# Supplementary material for: Comparing and integrating human mobility data sources for measles transmission modeling in Zambia
Source: PLOS Glob Public Health. 2025 May 20;5(5):e0003906. doi: 10.1371/journal.pgph.0003906 (PMC12091742; doi:10.1371/journal.pgph.0003906)

**S6 Fig. Posterior estimates of coefficients in exponential gravity model , using travel survey-fitted coefficients as a prior and mobile phone data (A) and Facebook data (B) as likelihood.** In both panels, the x-axis is the scaling factor by which the standard deviation of the coefficients of mobility model fit to travel survey was reduced by.


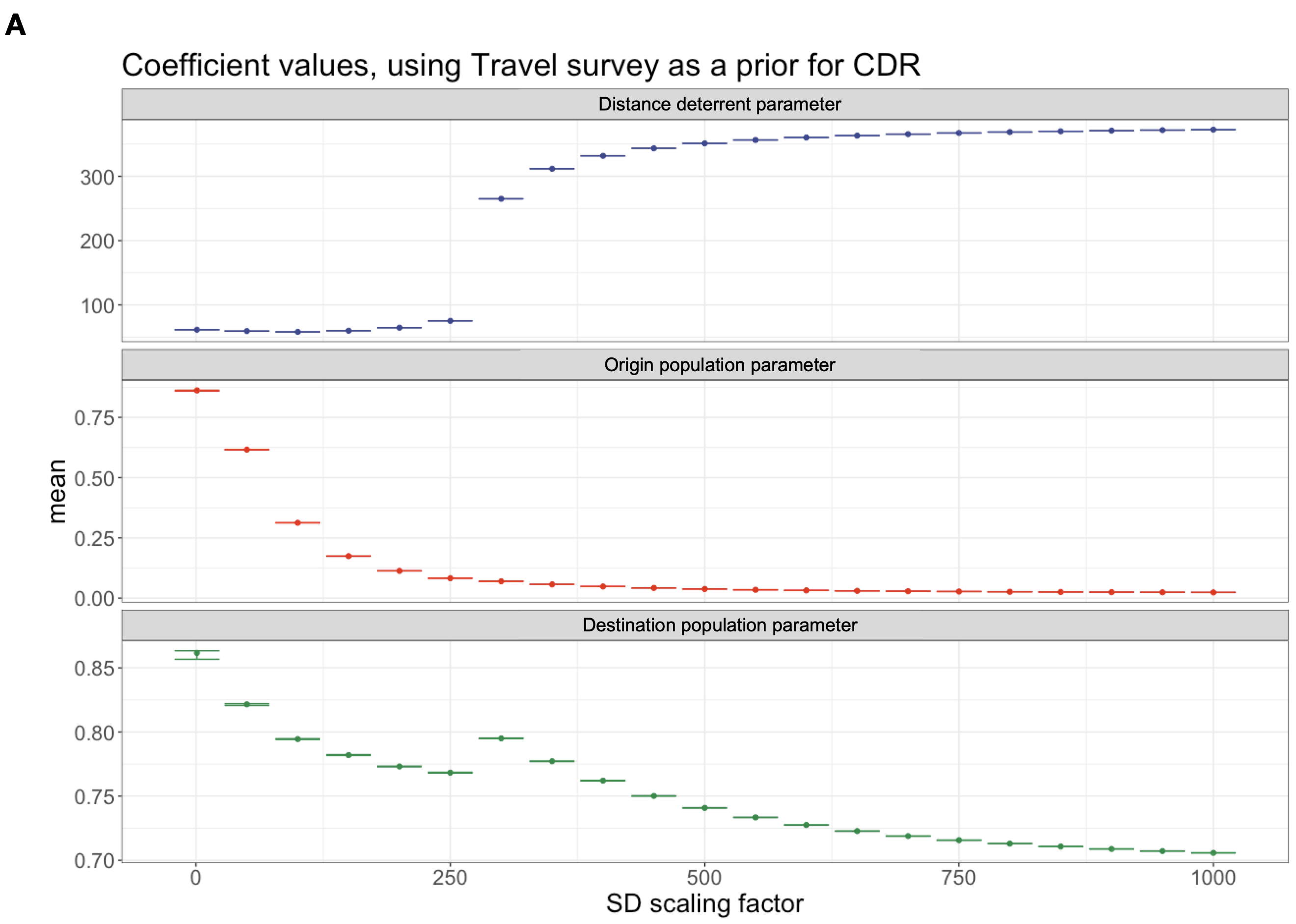


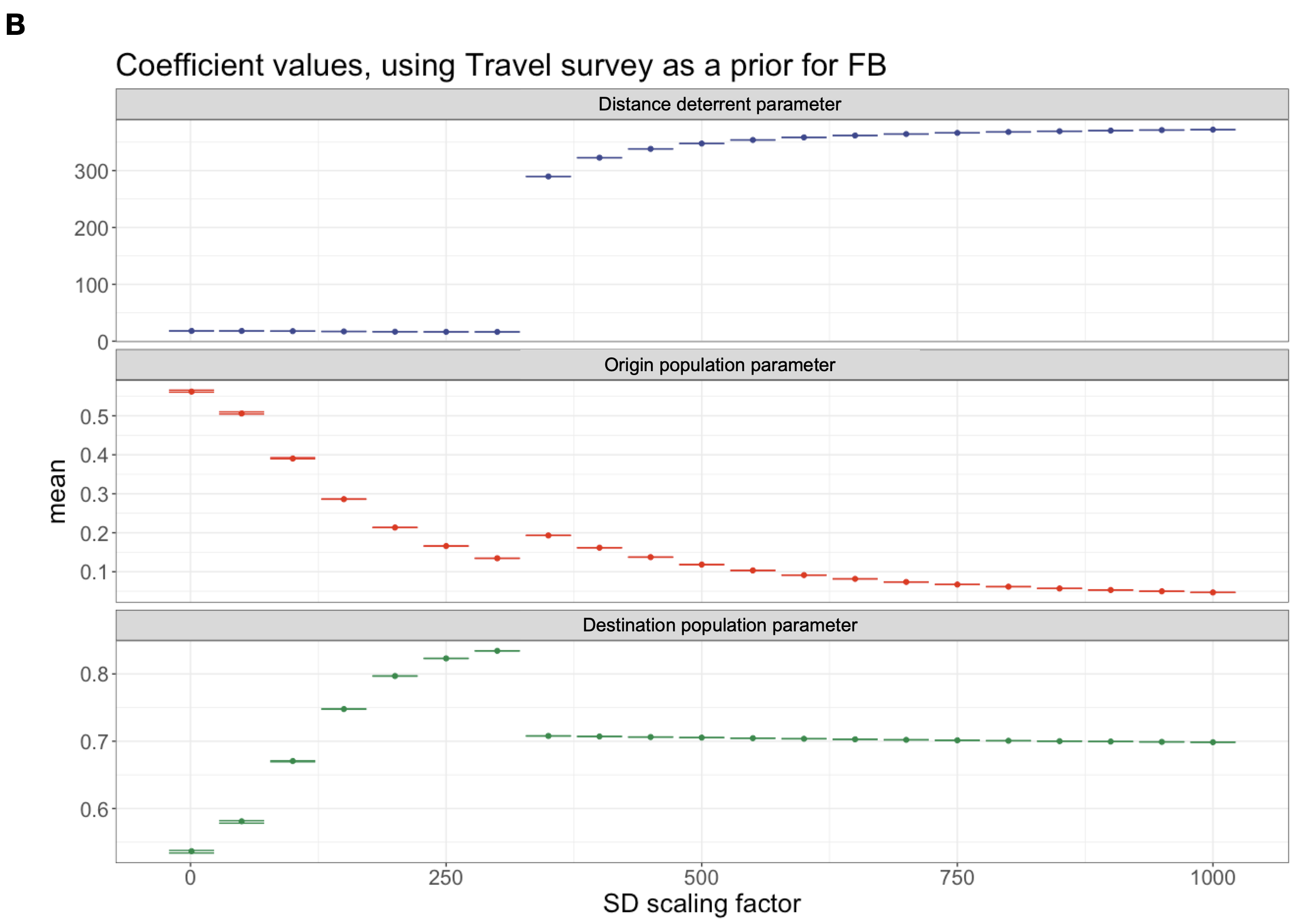

Supplement: S6 Fig — In both panels, the x-axis is the scaling factor by which the standard deviation of the coefficients of mobility model fit to travel survey was reduced by. (DOCX) [file pgph.0003906.s013.docx]
