# Supplementary material for: Comparing and integrating human mobility data sources for measles transmission modeling in Zambia
Source: PLOS Glob Public Health. 2025 May 20;5(5):e0003906. doi: 10.1371/journal.pgph.0003906 (PMC12091742; doi:10.1371/journal.pgph.0003906)
Supplement: Attachment — Submitted filename: main_manuscript (1).docx [file pgph.0003906.s014.docx]

Comparing different sources of human mobility data and methods of integration: modeling measles virus transmission in Zambia

Short title: Comparing and integrating different sources of human mobility data

Natalya Kostandova^1*^, Christine Prosperi^2^, Simon Mutembo^2^, Chola Nakazwe^3^, Harriet Namukoko^10^, Bertha Nachinga^3^, Shengjie Lai^7^, Andrew J Tatem^7^, Qianwen Duan^7^, Elliot N Kabalo^8^, Kabondo Makungo^9^, Gershom Chongwe^4^, Innocent Chilumba^11^, Gloria Musukwa^5^, Kalumbu H Matakala^12^, Mutinta Hamahuwa^12^, Webster Mufwambi^13^, Japhet Matoba^14^, Irene Mutale^11^, Kenny Situtu^11^, Edgar Simulundu^5^, Phillimon Ndubani^5^, Alvira Z Hasan^2^, Shaun A Truelove^1,2^, Amy K Winter^6.15^, Andrea C Carcelen^2^, Bryan Lau^1^, William J Moss^1,2^, Amy Wesolowski^1^

^1^Department of Epidemiology, Johns Hopkins Bloomberg School of Public Health, Baltimore, MD, 21205, USA

^2^Department of International Health, International Vaccine Access Center, Johns Hopkins Bloomberg School of Public Health, Baltimore, MD, 21205, USA

^3^Information, Research and Dissemination, Zambia Statistics Agency, Lusaka, Zambia

^4^Tropical Diseases Research Centre, Ndola, Zambia, PO Box 71769

^5^Administration, Macha Research Trust, Choma, PO Box 630166, Zambia

^6^Department of Epidemiology and Biostatistics, University of Georgia, Athens, GA, USA

^7^WorldPop, School of Geography and Environmental Science, University of Southampton, Southampton, UK, S017 1BJ

^8^Zambia Information and Communications Technology Authority, Lusaka, Zambia

^9^Zamtel, Lusaka, Zambia

^10^Population and Social Statistics, Zambia Statistics Agency, Lusaka, Zambia

^11^Biomedical Sciences Department, Tropical Diseases Research Centre, Ndola, Zambia, PO Box 71769

^12^Clinical Research Department, Macha Research Trust, Choma, PO Box 630166, Zambia

^13^Administration, Tropical Diseases Research Centre, Ndola, Zambia, PO Box 71769

^14^Molecular Biology Department, Macha Research Trust, Choma, PO Box 630166, Zambia

^15^Center for the Ecology of Infectious Diseases, University of Georgia, Athens, Georgia, USA

*Corresponding author: Natalya Kostandova, Department of Epidemiology, Johns Hopkins University, MD, 21205, USA**,** [nkostan1@jh.edu](mailto:nkostan1@jh.edu)

**Abstract**

Quantifying population mobility is crucial in developing accurate models of infectious disease dynamics. Increasingly, multiple data sources are available to describe individual and population mobility in a single location; however, there are no methods to systematically integrate these data. Combining information from these data sets may be valuable and help mitigate inherent biases in each data set due to sampling, censoring, and recall. We examined four commonly used data sources (mobile phone records, travel survey, Demographic and Health Survey, and Facebook location information) to quantify subnational travel patterns in Zambia. Estimates of the probability of a trip (departure) and the frequency of destination locations (diffusion) varied across data sets, with some data sets (mobile phone data, Facebook mobility data) estimating twice the frequency of travel as others (Demographic and Health Survey, travel survey). We developed a departure-diffusion model within a Bayesian framework to combine these data sets and jointly estimate travel, for which data from one data set served as a prior when fitting the model to another data set. When multi-data set models included mobile phone records, this data source dominated estimates given the broader spatial coverage. Based upon a metapopulation model to simulate a measles outbreak, using travel survey data to parameterize mobility resulted in the introduction of cases in 98% of districts compared to less than 50% when mobile phone data or Facebook data were used. This study highlights the need for methods that facilitate integrating multiple data sets to improve validity of mobility estimates and resultant infectious disease transmission dynamics.

**Introduction**

Population mobility drives the spread of infectious diseases and plays an important role in introducing pathogens into susceptible populations and maintaining transmission for a wide range of pathogens (1–16). Multiple data sources have been used to quantify human mobility, including surveys, mobile phone data (e.g. call detail record), Global Positioning System (GPS) loggers, transportation flows (e.g. airline, railroad, sea, and subway), national censuses, and online social network data (16–20). Often, a single mobility data source has been used despite spatial, temporal, and population differences across these data. For instance, national population-based household surveys like the Demographic and Health Surveys (DHS) are designed to be representative of the population of interest but are collected at infrequent temporal intervals. National population and housing censuses are intended to capture mobility data from all individuals within a country, but also at infrequent (often decennial) intervals. In contrast, call data records from mobile phones only measure the movement of mobile phone subscribers, which can lead to sparse coverage, especially in rural areas, but can do so on fine temporal and spatial scales. Data sets also vary by the type of travel quantified. For example, travel surveys traditionally rely on participants’ recall of trips taken, often over a long period, whereas mobile phone data has been used to quantify shifts in mobile phone tower usage by subscribers that serve as a proxy for travel. Overall, variation in these characteristics across data sets may result in different estimates of mobility due to differences in the sampled population, measurement method, and quantified travel type.

Increasingly, diverse mobility data sources have become available in the exact geographical location. This provides an opportunity to describe mobility and, importantly, identify how different data sets may impact estimates of spatial patterns of disease transmission. In addition, this may enable data sets to be adjusted to account for biases or be otherwise combined to obtain an integrated data set with more accurate or higher spatial or temporal coverage. While the availability of multiple data sources provides an opportunity to leverage unique information provided independently (21), challenges persist in accounting for disparate spatial and temporal scales and characteristics of individuals captured within each data set.

We assessed the comparability between estimates of human mobility across districts in Zambia from four commonly analyzed data sources: the 2018 Zambia DHS, call detail records obtained from a major mobile phone network provider, Facebook mobile application data, and an individual-level travel survey implemented in two districts. We used pooling and a Bayesian framework to combine information across datasets and assessed to what extent using these different data sets to quantify mobility in a transmission model affected estimates of introduction events using measles virus transmission as an example.

**Results**

First, we present a summary of the four data sets used in this analysis, specifically the type of mobility information captured and the population included (when available). Next, we provide a summary of the probabilities of departure at the district-level across the four data sets as well as the spatial coverage of information about departure. We then present results of three approaches using information from multiple data sets. First, we present results of using DHS data to adjust estimates of departure in mobile phone and Facebook data accounting for mobile phone ownership and internet use within the last 12 months. Second, we pool information on departure across all four data sets. Third, we present the results of a Bayesian framework in which model fit from one data set was used as a prior for fitting models to another data set, and thus provide a structure for developing single modeled outputs integrating multiple sources of mobility data. Next, we compare the coefficients from a departure-diffusion model fit to data from these three data sets with diffusion information. Finally, we present the results of simulations of measles virus transmission in Zambia, assessing the robustness of measures of measles virus introduction to the choice of data set used to inform mobility.

***Data sets measured different components of individual and population-level mobility:*** We analyzed four types of mobility data: community-based travel survey from two districts (2022), national multi-stage sampled DHS (2018), changes in locations of Facebook application users (4/2020-5/2022), and mobile phone data from a leading operator (3/2020-12/2020) (see Materials and Methods). The two individual-level data sets (travel survey and DHS) collected responses about overnight travel in the past two months (travel survey) and 12 months (DHS), but only the travel survey included the destination location. As the DHS interviewed women aged 15-49 years and men aged 15-59 years, the median age of respondents was 27 years old (IQR [20; 37]), with 53% of respondents women. The travel survey used stratified sampling to enroll caregivers of young and older children, as well as other adults in the home at the time of the survey; as a result, the sample was predominantly female (83%), with slightly older participants (median age 34 years; IQR [26; 43]; maximum age 96 years). The two population-level data sets (mobile phone data and Facebook mobility data) both included spatially and temporally aggregated flows between districts, with no information available about individuals in the data sets (see SI Appendix Table 1).

The mean probability of departure, calculated on a monthly scale for all datasets except Facebook (see Materials and Methods), was similar across data sets, ranging from 4% (DHS and travel survey) to 8% (mobile phone and Facebook data). The mobile phone and Facebook data sets were the most strongly correlated (Spearman’s correlation coefficient 0.529, p-value=0.045) (Fig 1C, SI Appendix Fig. S2A, SI Appendix Table S2). The probability of departure was generally highest in mobile phone data (median = 5.8%, IQR [4.2%; 8.4%]), followed by Facebook data (median = 4.5%, IQR [0.6%; 21.8%]), travel survey (3.7% and 4.3%), and DHS (median = 3.8%; IQR [3.0%; 4.3%]) (SI Appendix Fig. S2B-C). The probability of departure was 0 for most districts in the Facebook data set, notably for all the least populated districts (<112,000 people in 2020, 48/115 districts) and were excluded from additional analyses (SI Appendix Fig. S1B).

**Fig 1.** Coverage of the origin and destination districts in mobility data sets and the probability of travel from districts. A. Geographic coverage of districts of origin for each of four mobility data sets included in analysis. A district is said to be covered if there was a non-zero probability of travel reported from that district. B. Geographic coverage of destination districts for each of four mobility data sets included in analysis. A district is said to be covered if there were a non-zero number of trips reported into that district from another district. Destination information was not collected in the Demographic and Health Survey. C. Histogram of district-level probabilities of departure (travel) out of districts. Dashed line represents the mean probability of departure observed in each mobility data set.

The largest range of differences across data sources was in spatial coverage. For example, there were between 2 – 112 (2% - 97%) districts represented as origin locations for which the probability of departure was quantified (DHS = 112, mobile phone data = 107, Facebook data = 16, travel survey = 2) (Fig 1A) and between 0 – 107 as destination locations (DHS = 0 (no information on destination collected), mobile phone data = 107, Facebook data = 19, travel survey = 39) (see Fig 1B; SI Appendix Fig. S1).

***Adjusting for heterogeneity in the probability of overnight travel by mobile phone ownership and internet use from the DHS resulted in a decrease in the probability of departure in mobile phone and Facebook data****:* To assess the potential for sampling bias, based on mobile phone ownership for the mobile phone data and internet usage for the Facebook data set, we explored heterogeneity in travel by mobile phone ownership and internet use as reported in the DHS. We note that internet use was assessed using a single question on the DHS that ascertained any internet use in the last 12 months. The district-level probability of overnight travel in the DHS was lower for individuals who did not own a mobile phone or did not use the internet (Fig 2A-B) compared to mobile phone owners. Adjusting for ownership (mobile phone) and internet use (Facebook) biases from the DHS data resulted in a lower estimated probability of departure in 94% of districts for both Facebook and mobile phone data. The median change in probability of departure between unweighted and weighted estimates was -12% for mobile phone data (IQR [-22%; -3%]), and -39% for Facebook data (IQR [-100%; -8%]) (Fig 2C). Notably, weighting reduced the probability of departure to 0 for some of the districts (-100% change).

**Fig 2.** Accounting for heterogeneity in the probability of travel by mobile phone ownership and internet use in the last 12 months. A. Histogram of district-level probabilities of departure in the Demographic and Health Survey, stratified by reported mobile phone ownership. B. Histogram of district-level probabilities of travel in the Demographic and Health Survey, by district, stratified by reported internet use in the last 12 months. C. Percent change in probability of travel for each district following weighting by heterogeneity in travel by mobile phone ownership (mobile phone data set) and internet use (Facebook), as measured in the Demographic and Health Survey. Each point represents a district.

***Pooling probabilities of departure allowed for combined information on departure across data sets:*** Given the spatial sparsity of data on departure probabilities for the travel survey and Facebook data, we used pooling to obtain a single estimate of departure for each district using all four data sets. We first fit a beta-binomial model for the probability of departure from districts with available data and used this model to predict the probability of departure for all 115 districts in Zambia, regardless of coverage by each data set. We calculated pooled estimates of the probability of departure for each district using a random effect and inverse probability meta-analysis (see Materials and Methods). Compared to individual data set estimates, the pooled estimates generally had smaller variance than Facebook data and travel survey estimates, except for a few districts where the mobile phone estimate greatly differed from the DHS and Facebook estimate (see SI Appendix Fig. S3A, B). The pooled estimates also consistently decreased the departure probability compared to the raw mobile phone or Facebook data (SI Appendix Fig. S3A, B). Pooling weighted of the mobile phone and Facebook data sets alongside DHS and travel surveys consistently resulted in a lower probability of departure than pooling unweighted data (see SI Appendix Fig. S3C).

***Distance played a smaller role in the diffusion process estimated from the travel survey although estimates from the travel survey were less precise.*** To estimate travel across Zambia, we first fit an exponential gravity model to each data set (see SI Appendix for goodness of fit metrics from a range of spatial interaction models). Parameter estimates were consistent between the mobile phone and Facebook data sets with only the travel survey suggesting a considerably different parameter for the lower probability of travel with increasing distance (distance deterrent parameter). That is, from travel survey data, travel between two locations diminishes at a slower rate as distance increases, compared to other data sets. To leverage information across data sets simultaneously, we fit combined Bayesian exponential gravity models where the coefficient estimates from a model fit to one data set were used as a prior in the fit for a model from another data set. In general, given the imprecise nature of the fitted coefficients for the travel survey data, using this information as a prior did not substantially change the posterior distributions of model coefficients from either the mobile phone or Facebook data sets. The only exceptions were the two population size parameters, where using either travel survey or mobile phone data as a prior decreased the posterior estimates from the Facebook data (Fig 3). Conversely, models fit to travel survey data were sensitive to using either Facebook or mobile phone data as priors. The model coefficients fit to mobile phone data were more precise and, as a result, using Facebook coefficients as a prior did not shift the posterior (Fig 3). In these combined approaches, a single data set would need to be substantially more precise to serve as an informative prior when using data sets with higher spatial coverage (e.g. mobile phone data) (see SI Appendix Fig. S6).

Fig 3. The coefficient estimates for distance deterrence, origin population size, and destination population size parameters, using different combinations of sample data sets and priors. Distance deterrence parameter indicates the extent to which probability of travel decreases with increase in distance between origin and destination locations. Results are split into columns by the prior used (no prior, mobile phone data set prior, Facebook prior, and travel survey prior), and color-coded by which data set was used as a sample. The panel in the top-most left quadrant shows estimates for destination population coefficient when no prior data set was used; i.e., mobility model was fit to a single data set at a time (Facebook, mobile phone, or travel survey). The topmost panel in the second column shows estimates for the same coefficient (destination population) when a model was fit using mobile phone data as a prior, and either Facebook data or travel survey as the sample.

***Assessing the robustness of measles simulations to different estimates of mobility***

We next explored how the choice of mobility data set impacted estimates of measles virus introduction events across districts in Zambia. Using a stochastic compartmental model, we simulated the introduction of measles cases into the most populated, capital district Lusaka District (see SI Appendix for sensitivity analysis with varying initially infected locations), with mobility between districts parameterized by different combinations of departure and diffusion processes (see Materials and Methods) (see SI Appendix Fig. S4). Although the final epidemic size was relatively similar (Fig 4A, SI Appendix Table S3), when a single data set (travel survey, Facebook, or mobile phone data) was used to inform departure and diffusion processes, there were marked differences in the proportion of districts with at least one introduction event. Using mobile phone data only resulted in 35% of districts experiencing introductions, compared to 42% of districts using Facebook data and 98% of districts using travel survey data (SI Appendix Table S3, Fig 4B). The relative rankings of whether an introduction would occur in a specific district was consistent across data sets (correlation range 0.76-0.91, SI Appendix Table S4), but ranks for the mean time to introduction were less correlated (correlation range 0.22-0.68). While Facebook and mobile phone data were still moderately correlated (Spearman’s rho = 0.68), the correlation between travel survey results and either Facebook or mobile phone data results was low (SI Appendix Table S4).

Fig 4. Results of simulations of measles transmission dynamics following the introduction of 10 infectious cases into Lusaka District. A. Cumulative cases of measles with mobility between districts informed by a single data set (mobile phone, Facebook, or travel survey). B. Proportion of districts with introduction events with mobility between districts informed by a single data set. C. Proportion of districts with introduction events with results split by which data set was used to inform the diffusion process.

We next assessed whether combining information on diffusion and departure processes across combinations of data sets and pooled estimates affected simulated measles virus transmission dynamics. The probability of introduction remained below 40% if mobile phone data were used to inform the diffusion process, regardless of which data set was used to inform departure (weighted mobile phone data, DHS, pooled weighted or pooled raw) (Fig 4C, SI Appendix Table S3). When Facebook data were used to inform diffusion, however, the probability of introduction was more sensitive to the choice of the departure data set, falling below 40% when DHS data or pooled estimates were used (SI Appendix Table S3, Fig 4C). When diffusion was informed by the travel survey data, the results were most sensitive to the choice of departure data set, with less than 40% of districts having introductions when pooled estimates were used (SI Appendix Table S3, Fig 4C). Notably, less than 40% of districts had introduction events when pooled estimates were used for departure, regardless of which data set was used to inform the diffusion process (SI Appendix Table S3, Fig 4C). Use of travel survey data as a prior and Facebook data to inform likelihood did not change the proportion of districts with introduction events compared to using an uninformative prior (Fig 4C).

Finally, given the predominant use of a single data set to quantify mobility, we considered a population composed of one subgroup whose mobility had travel patterns described by the mobile phone data and a second subgroup whose mobility was best described by the travel survey. At least half of the population would have to be in the second subpopulation for the proportion of districts with introduction events to change by at least 25%. However, confidence intervals on the proportion of districts with introduction events increased as the proportion of the subpopulation with mobility described by the travel survey also increased (SI Appendix Fig. S7).

**Discussion**

As more data quantifying human mobility become available, it becomes increasingly important to understand the biases and limitations of these data sets to capture the processes of departure and diffusion. Inconsistency in mobility measures obtained from different data sources may have significant implications on infectious disease transmission models, especially as these models are used to classify spatial risk for diseases like measles. We compared mobility measures across Zambia from four commonly used data sets and evaluated the impact of using these data sets on estimates of simulated measles virus transmission across the country. The four data sets we examined - DHS, travel survey, mobile phone data, and Facebook data – differed in terms of geographic coverage, population demographics, and type of movement quantified. Both the departure and diffusion processes varied widely across data sets, with mobile phone and Facebook data generally estimating a higher proportion of travel out of district compared to DHS and travel survey data. Using these data sets in a measles virus transmission model resulted in different estimates of the proportion of districts with measles virus introduction events across the country, suggesting that the choice of mobility data set has significant implications for informing disease control programs.

The range in spatial coverage of each data set made it difficult to perform systematic comparisons of how they differed and methods to combine data sets. The time frame during which mobility was measured also varied, with DHS taking place before the COVID-19 pandemic. However, other studies show that while mobility in Zambia decreased during periods of travel restrictions, it returned to baseline levels within a few months of the start of the pandemic (22). Due to the much larger number of locations represented in the mobile phone data, this data set dominated the posterior in the Bayesian approach to modeling the diffusion process. On the other hand, the coefficients estimated from fitting the mobility models to travel survey data would have to be much more precise to serve as an informative prior when combined with mobile phone or Facebook data. This level of precision may not be feasible by increasing the sample size of the travel survey alone and, given the large disparities between these data sets, understanding how informative travel captured by the travel survey compares to other data sets with wider spatial coverage would need to be determined. When no information about the relative importance of data sets exists, pooling estimates across data sets can be a useful option.

The observed differences in estimates of both departure and diffusion processes across data sets included in this study are concerning, especially as a single data set is usually used to quantify mobility in models of disease transmission. The use of mobile phone and Facebook data resulted in a highly correlated ranking of districts based on probabilities of introduction, which could provide confidence in the allocation of resources to different districts. However, the correlation was not consistently high for ranks of the introduction time. While data sets like mobile phone data have high geographical coverage, capture variation in travel over time, and can be made available almost in real-time, additional studies must be undertaken to quantify travel differences among individuals included in mobile phone data and those who are excluded. Ultimately, more refined estimates of human mobility can improve our understanding of spatial patterns of disease transmission and allow for shaping more effective disease control programs. However, in the meantime, nationwide surveys like DHS or the Zambia Population-based HIV/AIDS Impact Assessment could integrate a travel survey module to provide a representative sample of the population, such as the movement of children, who are missed from many other data sets but are important to the transmission of many pathogens. Additionally, metadata included in population-level data sets, such as gender, age, and socio-economic status of individuals for whom data were collected, could help quantify the direction and magnitude of bias. Leveraging the information available across different sources of mobility data has the potential to adjust parameter estimates, improve geospatial and temporal coverage of data, and better capture travel patterns of populations.

**Materials and Methods**

***Mobility data sets:*** We analyzed four data sets describing human mobility patterns across Zambia (see SI Appendix Table 1, SI Appendix Fig. S1).

***Travel survey***: A travel survey was nested in a serological survey of IgG antibodies to measles virus in two districts in Zambia (Choma and Ndola) from March – June 2022 (23). In total, 3577 individuals across 72 clusters and 1245 households were surveyed. The survey collected information on socio-demographic characteristics of participants, mobile phone ownership, and overnight travel in the prior two months, including the number of trips taken, district(s) traveled to, the reason for travel, and whether the respondent was accompanied by adults or children. To obtain the number of monthly trips taken between districts of origin in Choma or Ndola and other districts, the reported number of trips between each pair of origin and destination districts taken in the last two months was divided by two.

***Demographic and Health Survey (DHS)***: The most recent Zambia DHS was carried out in 2018 and was implemented using a stratified two-stage sample design (24). See (24) for a full description of the DHS methodology and questionnaires. In this analysis, the relevant questionnaire modules included socio-demographic characteristics of participants, mobile phone ownership, internet use within the last 12 months, and overnight travel in the last 12 months. Using the jittered GPS coordinates per household, each respondent household was matched to the corresponding district. A sampling-weighted estimate of overnight travel in the last 12 months was obtained for each district and divided by 12 to produce the average monthly probability of at least one overnight trip.

***Mobile phone data:*** A spatially and temporally aggregated data set of travel between districts was obtained from the Zambia Telecommunications (Zamtel) Company Limited, one of the primary phone operators in Zambia. Based on number of daily subscribers provided by Zamtel during this period, the highest number of subscribers on a single day corresponds to 10.5% of 2020 population of Zambia, ranging from 0% to over 40% in some districts. From March 1, 2020, to December 30, 2020, the number of Zamtel anonymized SIM IDs for which the primary location changed from one district to another between subsequent days was recorded. SIM IDs with primary cell tower locations in the same district for subsequent days were considered to have not left the district (“stay”). Cell towers were aggregated to the district level using the 2018 administrative boundaries. Due to temporal fluctuations in travel during the study period, including changes in restrictions during the COVID-19 pandemic, we further aggregated mobile phone data to obtain the total number of stays and the total number of trips between districts over the nine-month period.

***Facebook data at Meta:*** The mobility data of Facebook users during the COVID-19 pandemic were obtained from April 1, 2020, through May 21, 2022. These data were limited to mobile devices with the Facebook application installed that had opted to share geolocation. Details of this data set are available elsewhere (25,26). Data were aggregated spatially to Bing tile level 12 by Meta, approximately 9.8 km at the equator (27). For each 8-hour period, the number of individual devices that moved from one tile to another across adjacent 8-hour periods was collected. The user’s location was assigned to the tile in which they appeared most frequently during each 8-hour window, or to the last tile if the frequency was the same. In the tile-level data set made available for this study, the number of individuals moving from one tile to another was discarded if fewer than ten trips were observed. Data were then aggregated to the district level, so individuals who remained in the same district across adjacent 8-hour periods were considered a “stay”. To account for potential fluctuations in travel due to the COVID-19 pandemic, we aggregated the Facebook mobility data to obtain the total number of stays and the total number of trips taken between districts over the period reported.

***Weighting mobile phone and Facebook data to account for sampling bias:*** Using the DHS data, we calculated a sampling-weighted probability of departure by district stratified by phone ownership or internet use, as well as the proportion of individuals who owned a mobile phone and/or reported using the internet. We then used these estimates to weight the mobile phone and Facebook mobility data sets to better capture travel by individuals who did not have a mobile phone and did not use the internet in the last 12 months, respectively (see *SI Appendix)*. We recognize that this is likely to only partially account for sampling bias, especially as the DHS does not capture use of Facebook app on smartphone, which is a requirement for being included in the Facebook mobility data.

***Fitting the probability of departure***: We calculated the probability of departure (i.e., a trip outside of the origin district) per district for each data set using the monthly average or estimated value. For each of the four data sets, we independently fit a hierarchical beta-binomial model using the *mobility* package in R (28) (*SI Appendix*).

***Obtaining pooled estimates for the probability of departure:*** We pooled fitted estimates for the probability of departure from all four data sets in either the raw (DHS, travel survey, raw mobile phone, raw Facebook) or weighted (DHS, travel survey, weighted mobile phone, weighted Facebook) forms. We used a random-effect model to account for differences in the time and population captured by these data sets. Pooling was done with the inverse variance method using the *meta* package in R (29). For each district, we obtained a point estimate and standard error for the pooled probability of departure.

***Modeling diffusion patterns:*** We analyzed how the various data sets performed in estimating the destination district for a trip, given that an individual departed their district of origin. This analysis excluded the DHS data since it did not collect information on the travel destination. We first fit gravity models with different structures and selected a best-fit model for each data set. For the best-fitting model selected, we compared coefficient estimates across the three data sets (see *SI Appendix*).

To incorporate information on mobility from more than one data set, we used a Bayesian framework. We used estimated coefficients and posterior distributions from the model fit to one data set as a prior for fitting the model to another data set. We modified the source code of the *mobility* package to include an informative prior, where population and distance parameter priors for travel between two districts were drawn from a truncated normal distribution with mean and precision set to estimates of coefficients from the diffusion model fit to another data set. Models were implemented using *rjags* library in R (30).

To evaluate how precise coefficient estimates from a model fit to one data set must be to be an informative prior, we reduced the standard deviation on the coefficients by a single factor. We then drew values of coefficient from distributions with fitted mean and the scaled standard deviation and used those as a prior in the Bayesian approach. The second data set used in the Bayesian framework as the sample remained unchanged.

***Evaluating the impact of various mobility matrices informed by each data set on measles virus transmission:*** We constructed a discrete-time spatial stochastic model of measles virus transmission. We focused on measles as a case study due to the well-understood transmission dynamics, low levels of community transmission in Zambia prior to 2020, and interest of the Zambia Ministry of Health on identifying subnational areas for targeted interventions like Supplementary Immunization Activities.

Briefly, in our model, individuals transitioned between Maternal Immunity, Susceptible, Infected, Vaccinated, and Removed compartments. We seeded 10 infections in Lusaka District where the capital city is located (10 to avoid too many stochastic fadeouts). We ran 100 simulations for 1 year to capture the early dynamics of an outbreak across Zambia. Details of the transmission model and initial parameters are provided in *SI Appendix*.

For each mobility matrix derived from the various data sources and their combinations, we calculated the proportion of districts with an introduction event, defined as having the mean number of cumulative infections greater than or equal to one. We also calculated the mean number of cumulative cases over the course of 1 year and the 2.5th and 97.5th quantiles of cumulative cases nationwide.

As sensitivity analysis, we conducted simulations of measles virus dynamics following the introduction of 5 cases each in Ndola District in northern Zambia and Choma District in southern Zambia. We used 5 cases in each of the two districts to seed the same number of total infections as in the main scenario (10) but distributed between the two districts due to their lower connectivity compared to Lusaka District.

***Identifying what proportion of the population would have to be described by the travel survey to result in different estimates of introduction events than using mobile phone data to quantify population mobility:*** In this approach, a portion of the population had mobility patterns described by the mobile phone data and the remaining proportion of the population described by the travel survey. We selected these two data sets as mobile phone data had the highest spatial coverage in Zambia while the travel survey had the lowest. We scaled the standard deviation on travel survey-fitted coefficients so that precision relative to the mean was equivalent to that of the mobile phone data-fitted coefficients. We varied the proportion of population described by mobile phone data from 0 to 1, obtained a weighted average of the mobile phone-informed and travel survey-informed mobility matrices, and carried out simulations as described above using the weighted mobility matrix. Simulations were repeated 100 times and the proportion of districts with introduction events was obtained as previously described.

**Ethics statement**

This study was submitted to the Johns Hopkins Bloomberg School of Public Health Institutional Review Board and was found to be Not Human Subjects Research (FWA #00000287), as it relies on use of existing, de-identified data collected prior to this study.

**Acknowledgments**

We are grateful to the communities that welcomed the travel survey teams, and to the participants who kindly shared their experiences and their time with us. The authors would like to also acknowledge the Data for Good program at Meta for sharing data and thank Dr. Fatumah Atuhaire for Facebook mobility data collation.

**References**

1.	Nguyen THT, Nguyen TV, Luong QC, Ho TV, Faes C, Hens N. Understanding the transmission dynamics of a large-scale measles outbreak in Southern Vietnam. Int J Infect Dis. 2022 Sep;122:1009–17.

2.	Parpia AS, Skrip LA, Nsoesie EO, Ngwa MC, Abah Abah AS, Galvani AP, et al. Spatio-temporal dynamics of measles outbreaks in Cameroon. Ann Epidemiol. 2020 Feb;42:64-72.e3.

3.	Khatua A, Kar TK, Nandi SK, Jana S, Kang Y. Impact of human mobility on the transmission dynamics of infectious diseases. Energy Ecol Environ. 2020;5(5):389–406.

4.	Mari L, Bertuzzo E, Righetto L, Casagrandi R, Gatto M, Rodriguez-Iturbe I, et al. Modelling cholera epidemics: the role of waterways, human mobility and sanitation. J R Soc Interface. 2012 Feb 7;9(67):376–88.

5.	Finger F, Genolet T, Mari L, de Magny GC, Manga NM, Rinaldo A, et al. Mobile phone data highlights the role of mass gatherings in the spreading of cholera outbreaks. Proceedings of the National Academy of Sciences. 2016 Jun 7;113(23):6421–6.

6.	Wesolowski A, Eagle N, Tatem AJ, Smith DL, Noor AM, Snow RW, et al. Quantifying the Impact of Human Mobility on Malaria. Science. 2012;338(6104):267–70.

7.	Tatem AJ, Smith DL. International population movements and regional *Plasmodium falciparum* malaria elimination strategies. Proceedings of the National Academy of Sciences. 2010 Jul 6;107(27):12222–7.

8.	Carrasco-Escobar G, Matta-Chuquisapon J, Manrique E, Ruiz-Cabrejos J, Barboza JL, Wong D, et al. Quantifying the effect of human population mobility on malaria risk in the Peruvian Amazon. R Soc Open Sci. 2022 Jul;9(7):211611.

9.	West SK, Munoz BE, Mkocha H, Gaydos C, Quinn T. Risk of Infection with Chlamydia trachomatis from Migrants to Communities Undergoing Mass Drug Administration for Trachoma Control. Ophthalmic Epidemiol. 2015;22(3):170–5.

10.	Lurie MN, Williams BG, Zuma K, Mkaya-Mwamburi D, Garnett GP, Sturm AW, et al. The Impact of Migration on HIV-1 Transmission in South Africa: A Study of Migrant and Nonmigrant Men and Their Partners. Sexually Transmitted Diseases. 2003 Feb;30(2):149.

11.	Mmbaga EJ, Leyna GH, Hussain A, Mnyika KS, Sam NE, Klepp KI. The role of in-migrants in the increasing rural HIV-1 epidemic: results from a village population survey in the Kilimanjaro region of Tanzania. International Journal of Infectious Diseases. 2008 Sep 1;12(5):519–25.

12.	Metcalf CJE, Cohen C, Lessler J, McAnerney JM, Ntshoe GM, Puren A, et al. Implications of spatially heterogeneous vaccination coverage for the risk of congenital rubella syndrome in South Africa. J R Soc Interface. 2013 Jan 6;10(78):20120756.

13.	Gog JR, Ballesteros S, Viboud C, Simonsen L, Bjornstad ON, Shaman J, et al. Spatial Transmission of 2009 Pandemic Influenza in the US. PLOS Computational Biology. 2014 Jun 12;10(6):e1003635.

14.	Charaudeau S, Pakdaman K, Boëlle PY. Commuter Mobility and the Spread of Infectious Diseases: Application to Influenza in France. PLOS ONE. 2014 Jan 9;9(1):e83002.

15.	Yaméogo KR, Perry RT, Yaméogo A, Kambiré C, Kondé MK, Nshimirimana D, et al. Migration as a risk factor for measles after a mass vaccination campaign, Burkina Faso, 2002†. International Journal of Epidemiology. 2005 Jun 1;34(3):556–64.

16.	Barbosa H, Barthelemy M, Ghoshal G, James CR, Lenormand M, Louail T, et al. Human mobility: Models and applications. Physics Reports. 2018;734:1–74.

17.	Isdory A, Mureithi EW, Sumpter DJT. The Impact of Human Mobility on HIV Transmission in Kenya. PLOS ONE. 2015 Nov 24;10(11):e0142805.

18.	Kraemer MUG, Golding N, Bisanzio D, Bhatt S, Pigott DM, Ray SE, et al. Utilizing general human movement models to predict the spread of emerging infectious diseases in resource poor settings. Scientific Reports. 2019 Mar 26;9(1):5151.

19.	Lai S, Johansson MA, Yin W, Wardrop NA, van Panhuis WG, Wesolowski A, et al. Seasonal and interannual risks of dengue introduction from South-East Asia into China, 2005-2015. PLoS Negl Trop Dis. 2018 Nov;12(11):e0006743.

20.	Bengtsson L, Gaudart J, Lu X, Moore S, Wetter E, Sallah K, et al. Using mobile phone data to predict the spatial spread of cholera. Sci Rep. 2015 Mar 9;5:8923.

21.	Gootzen YAPM, Roos MR, Mussmann BO. Combining data sources to gain new insights in mobility: A case study. Center for Big Data Statistics; 2020 Jul. Report No.: Working paper no.: 03-20.

22.	Loisate S, Mutembo S, Arambepola R, Makungo K, Kabalo EN, Sinyange NB, et al. Changes in mobility patterns during the COVID-19 pandemic in Zambia: Implications for the effectiveness of NPIs in Sub-Saharan Africa. PLOS Glob Public Health. 2023 Oct 31;3(10):e0000892.

23.	Kostandova N, Prosperi C, Mutembo S, Nakazwe C, Namukoko H, Nachinga B, et al. Adjusting mobile phone data to account for children’s travel and the impact on measles dynamics in Zambia. American Journal of Epidemiology. (in press);

24.	Zambia Statistics Agency (Formerly Central Statistical Office). The DHS Program - Zambia: Standard DHS, 2018 [Internet]. 2020 [cited 2023 Feb 22]. Available from: https://dhsprogram.com/methodology/survey/survey-display-542.cfm

25.	Leasure DR, Kashyap R, Rampazzo F, Dooley C, Elbers B, Bondarenko M, et al. Nowcasting daily population displacement in Ukraine through social media advertising data [Internet]. SocArXiv; 2022 [cited 2023 Feb 16]. Available from: https://osf.io/preprints/socarxiv/6j9wq/

26.	Shepherd HER, Atherden FS, Chan HMT, Loveridge A, Tatem AJ. Domestic and international mobility trends in the United Kingdom during the COVID-19 pandemic: an analysis of facebook data. International Journal of Health Geographics. 2021 Dec 4;20(1):46.

27.	Microsoft. Bing Maps Tile System - Bing Maps [Internet]. 2022 [cited 2023 Feb 16]. Available from: https://learn.microsoft.com/en-us/bingmaps/articles/bing-maps-tile-system

28.	Giles J, Wesolowski A. mobility: Tools for analyzing human mobility data [Internet]. 2020. Available from: https://github.com/COVID-19-Mobility-Data-Network/mobility

29.	Balduzzi S, Rücker G, Schwarzer G. How to perform a meta-analysis with R: a practical tutorial. Evidence-Based Mental Health. 2019;(22):153–60.

30.	Plummer M. rjags: Bayesian Graphical Models using MCMC [Internet]. 2022. Available from: https://CRAN.R-project.org/package=rjags
