# Supplementary material for: Comparing and integrating human mobility data sources for measles transmission modeling in Zambia
Source: PLOS Glob Public Health. 2025 May 20;5(5):e0003906. doi: 10.1371/journal.pgph.0003906 (PMC12091742; doi:10.1371/journal.pgph.0003906)
Supplement: Attachment — Submitted filename: response_reviewers_20241016.docx [file pgph.0003906.s017.docx]

Response to the reviewers

We are grateful to the reviewers for their helpful feedback and suggestions.

We have made the following changes in the manuscript:

- Provided more information about datasets used and the consenting procedure in “Ethics statement”;
- Changed the order of sections so Materials and Methods come before presentation of Results;
- Expanded the discussion to explicitly state limitations of data sources used;
- Contextualized the study within the larger body of literature.

In addition, there were several comments in the article of sections in the results that recommended to move certain sentences to the materials and methods section. Given the large number of approaches and data sets used in the manuscript, we found that this guidance can help the reader orient themselves to the overall narrative. For example, there are multiple types of models used (e.g. mechanics, statistical including modeling mobility and transmission) which serve different purposes. A general statement of modeling results could confuse the reader on which approach was being used. We have edited a number of phrases to improve clarity in the text. However, we defer to the editor if they believe they should be consistently removed.

Reviewer's Responses to Questions

Reviewer #1: The manuscript titled, "Comparing different sources of human mobility data and methods of integration: modelling measles virus transmission in Zambia" is a credible innovative analysis. It however requires some minor review to meet PLOS Global Public Health's standards. The following major comments about the manuscript are stated below:
1. The manuscript lacks clear objective (the objective is expected to be SMART). Although it was written at the end of the introduction, it looks cumbersome and needs to be reviewed.

**Response**: Thank you. We’ve revised the objective statement to read as follows (lines 90-104): “In this study, we aimed to assess 1) the comparability between estimates of human mobility across districts in Zambia obtained from four data sources; 2) the feasibility of using pooling and a Bayesian framework to combine information from multiple datasets; and 3) the extent to which choice of the dataset or combination of datasets to quantify mobility affected estimates of introduction events using a measles virus transmission framework in Zambia as an example. To achieve these objectives, we first compared probabilities of departure and diffusion from the 2018 Zambia DHS, call detail records from a major mobile phone network provider in Zambia, Facebook mobile application data, and an individual-level travel survey implemented in two districts of the country. We then used pooling and a Bayesian framework to combine information across datasets; in the Bayesian framework, probabilities of departure and diffusion from one dataset were used as a prior when fitting probabilities of departure and diffusion from another dataset to obtain a posterior informed by both datasets. Finally, we used a meta-population compartmental model to simulate dynamics of measles virus across Zambia, and compared the estimates of introduction events occurring when different mobility datasets were used to inform population movement.”

2. The method of comparison and the automated meta-analysis of data needs to be clearly provided in the method section.

**Response:** In the methods section we have provided only descriptions of each data set to help orient the reader to differences in the spatial and temporal scales, methods of collection, and granularity. We did not conduct a formal meta-analysis of the data since they differed in a number of dimensions and believe that the descriptive results can provide a clearer understanding. However, we welcome additional feedback on possible comparisons that should be conducted.

We also updated reference to “’inverse probability meta-analysis” when describing pooling approach to read, “inverse probability weighting,” which was done using meta package in R. Additional information about this method is available in the reference provided.

3. Some descriptions of methods were found in the results section, and this is not acceptable.

**Response:** We have removed some sections as suggested, but we would prefer to keep some of the sentences flagged for moving to the methods section. We believe that they help orient the reader as to which results are being presented by reminding them which approach they stem from.

4. Some discussions were also included in the results section, and this is not in line with the manuscript's guideline

**Response:** Thank you, we have removed these as suggested by the reviewer in the manuscript file. However, there are a couple instances where the flagged sentences present / summarize what we observed from the data, so we prefer to keep as is in the Results section.

5. The Method section is supposed to come before the results.

**Response:** We have moved the Materials and Methods section before the results as suggested.

If these observed comments were considered by the authors, I would suggest that the manuscript should be considered for publication.

Reviewer #2: The manuscript titled Comparing Different Sources of Human Mobility Data and Methods of Integration: Modeling Measles Virus Transmission in Zambia presents a significant effort in analyzing the integration of human mobility data using a Bayesian framework to model measles transmission dynamics in Zambia. The study is innovative however following points should be addressed in the revised version to enhance the manuscript’s clarity, precision, and impact.

The introduction, while informative, requires strengthening to clarify the specific objectives of the study. The opening sentence referencing the first 16 citations lacks specificity regarding the modes of transmission studied, which range from fecal-oral to airborne and vector-borne. Providing these details would immediately set the stage for the relevance of the study within the broader context of infectious disease transmission modelling. Furthermore, recent studies that explore the use of human mobility data in the context of SARS-CoV-2, such as Berke A, Doorley R, Alonso L, Arroyo V, Pons M, Larson K. Using mobile phone data to estimate dynamic population changes and improve the understanding of a pandemic: A case study in Andorra. PLoS One. 2022 Apr 26;17(4):e0264860. doi: 10.1371/journal.pone.0264860, should be incorporated to reflect the most current advancements in the field. This would not only broaden the context but also underscore the applicability of the study's findings to contemporary public health challenges.

**Response:** Thank you, we have updated the text to read as follows, “Population mobility drives the spread of infectious diseases and plays an important role in introducing pathogens into susceptible populations and maintaining transmission for a wide range of pathogens (1,2), including measles (3–5), cholera (6,7), malaria (8–10), trachoma (11), human immunodeficiency virus (12,13), rubella (14), and influenza (15,16), among others. Use of different sources of data to quantify mobility has particularly increased since the beginning of the SARS-CoV-2 pandemic (17), with population mobility used to inform questions across different phases of public health response, including prior to an outbreak, during emergence, during outbreak, and elimination (18)” (lines 63-70).

The manuscript’s reference to the Global Positioning System (GPS) should be more precise, particularly in distinguishing GPS from the broader category of Global Navigation Satellite Systems (GNSS). While the term GPS is initially used correctly within the manuscript, later uses are confusing. It is essential to acknowledge that modern devices often utilize multiple GNSS systems, such as Galileo or GLONASS beyond GPS. This distinction is critical for accuracy, as it affects the interpretation of the data and its generalizability to environments with varying GNSS coverage, especially in light of ever-increasing GPS Spoofing. (A. Altaweel, H. Mukkath and I. Kamel, "GPS Spoofing Attacks in FANETs: A Systematic Literature Review," in IEEE Access, vol. 11, pp. 55233-55280, 2023, doi: 10.1109/ACCESS.2023.3281731)

**Response:** Thank you, we acknowledge that GPS is but one category of GNSS, and updated the section of introduction making reference to GPS to explicitly state that. Namely, lines 70-74 now read “Multiple data sources have been used to quantify human mobility, including, but not limited to, surveys, mobile phone data (e.g. call detail record), Global Positioning System (GPS) loggers and other Global Navigation Satellite Systems, transportation flows (e.g. airline, railroad, sea, and subway), national censuses, and online social network data (16–20).” We confirm that the other explicit reference to GPS is in the context of geo-coordinates collected during the 2018 ZDHS (“Using the jittered GPS coordinates per household, each respondent household was matched to the corresponding district.”). We confirm that the ZDHS documentation specifically refers to these data as GPS, so we believe this wording to be appropriate. We do not explicitly specify whether locations obtained from Facebook data were collected via GPS or other types of GNSS, but we have added a perspective in the discussion section acknowledging the implication of increased use of multiple GNSS as opposed to GPS.

On line 73, the limitations of call data should be more explicitly discussed. Studies have shown that in many regions, VoIP services like WhatsApp have overtaken traditional mobile phone usage due to the lower cost of data compared to voice calls. This shift can significantly impact the representativeness of the data. Studies have demonstrated that reliance on phone call data might omit substantial segments of the population who primarily use VoIP or have financial restraints in making mobile phone calls (doi: 10.1016/j.socscimed.2018.01.011 and doi: 10.1177/08944393221111340)

**Response:** We’ve expanded the discussion section to explicitly include discussion of these limitations, in addition to evidence that mobile phone ownership and use can be heterogeneous by different socio-economic and demographic characteristics. Namely, we have added, “Existing literature provides evidence that mobile phone ownership is different based on socio-economic and demographic factors like age, gender, and other characteristics (23–27). Previous analysis using the travel survey and mobile phone data used in this study found that travel varied by mobile phone ownership and by age (28); with young children less likely to own or carry mobile phones, their exclusion from mobile phone data is likely to result in biased estimates of travel. Furthermore, even among individuals that do own a mobile phone, financial restraints in making calls or sending a message (29), especially in presence of alternative options like WhatsApp (30), may result in exclusion from call detail records.” (lines 461-468)

The discussion starting at line 89 would benefit greatly from an in-depth review of prior work, particularly studies that have integrated multiple data sources for infectious disease modeling. (doi: 10.1080/17538947.2021.1886358) This would not only situate your work within the existing literature but also highlight the innovative aspects of your approach.

**Response:** We have included references to such studies in the discussion section, also highlighting instances where studies compared mobility patterns from multiple data sources. We’ve referenced two studies that provided frameworks from bringing together data from multiple sources, and agree this helps situate our study and findings within the larger context.

Line 289 and the subsequent discussion sections should explicitly address the limitations of the study, particularly concerning the mobile phone data. For instance, during the data collection period, Zamtel was operating on a 2G network in some regions, which may have affected the accuracy of location data due to the broader cell tower range in flatter terrains. This could result in devices being incorrectly located in a different district than where they actually were. A discussion of this limitation is necessary to temper the interpretation of the results.

**Response:** Thank you, we have added this limitation in discussion. Namely, we’ve stated, “In addition to sampling biases, mobility data collected using digital devices like mobile phones may also have varying accuracy depending on devices and characteristics of networks used in data collection. For instance, the area covered by a cell tower in areas with 2G network are larger than areas covered by 3G, 4G, and 5G networks. With 2G more common in rural areas of Zambia, it is likely that there was higher inaccuracy in travel origin and/or destinations when travel included at least one terminus in a rural area, potentially resulting in higher rate of misclassification of district of origin or destination.” (lines 478-484)

Furthermore, on line 315, it is important to acknowledge that a unique SIM card does not necessarily correspond to a mobile phone. Many IoT devices, such as vehicle trackers, also utilize SIM cards, which could skew the data if not accounted for. If the data set used in the study was filtered to include only mobile phone users, this should be clearly stated to avoid any potential misinterpretation.

**Response:** We have included this point in the discussion. In addition, we added a mention that a single SIM card may not map to a single individual; for example, our previous work found that caregivers of young children under 5 often traveled with children. A single SIM card in that case would capture travel of multiple individuals. The paragraph in discussion now reads, “On the other hand, travel recorded by a unique SIM card may not directly map to travel by a single individual. Some individuals may own multiple SIM cards; others may travel with children or other individuals that do not own a mobile phone or a SIM card from the same network provider. For example, in the travel survey, caregivers of children under 5 years of age reported that when they undertook an overnight trip, they traveled with on average 0.48 children in Choma and 0.59 children in Ndola (28). SIM cards used in Internet of Things devices may also be captured in call detail records, but would capture a different dimension of travel than SIM cards in mobile phones, especially if used in long-haul vehicles. The extent to which this could bias departure and diffusion processes captured by mobile phone data is likely to vary across settings.” (line 468-477)

The Materials and Methods section would benefit from a more detailed discussion of the limitations of the various data sources used. For instance, the potential for recall bias in the travel survey data is significant and should be addressed.

**Response:** Thank you, we’ve added a more thorough section discussion potential limitations of the data sources. Namely, we explicitly acknowledge differential ownership of mobile phones by different socio-economic characteristics, with particular focus on under-representation of young children from mobile phone data. We also incorporated a discussion of recall bias in the travel survey, and acknowledged the limitation of travel surveys in capturing seasonal and other temporal fluctuations in travel.

Additionally, the explanation of the Bayesian framework should be expanded. It is currently not perfectly clear how priors were selected and how they influenced the posterior distributions, which is particularly important for readers who may not be well-versed in Bayesian methodologies.

**Response:** We focused the manuscript on the main findings of this work, not necessarily the multiple types of fitting approaches that were explored in the initial mobility package. We have included additional details in the supplement (*Combining information from two datasets using a Bayesian framework)* since this was not the main focus on the paper.

The justification for the random-effects approach, although sound, could also be expanded with reference to foundational statistical texts, such as Gelman et al. (2004) (doi: 10.1214/009053604000001048), to provide a more rigorous defense of this choice.

**Response:** We have now included this reference, and more explicitly stated that our datasets likely capture information about different populations due to differences in time, geography, and sampling strategies.

The brief mention of the SARS-CoV-2 pandemic’s impact on mobility patterns requires further elaboration. Specifically, a discussion on how the pandemic may have affected the validity of the data and whether any novel methods could account for these sudden and drastic changes would add substantial value to the manuscript. This could include, for example, the potential for real-time data correction methods or the use of alternative data sources during periods of lockdown or reduced mobility.

**Response:** In our data sets, for those collected over the period of the COVID-19 pandemic, they consistently demonstrated an impact on travel and hence it would be difficult to use one of these data sets in lieu of another if the impact on travel was systematically observed. In our analyses, data not collected during the pandemic would obviously, not be impacted and could be used in a joint model to provide estimates of mobility. However, in many instances during a public health emergency the primary question of interest is how mobility is changing. Other data sets, over different time frames, could be used to estimate a baseline mobility pattern. However, these would be subject to biases since they temporally mismatch the time period of interest. We have now included additional text discussing the COVID-19 pandemic’s impact on mobility in Zambia (using a subset of these data) to help further illustrate this point. We have now added this line to the discussion: “The differences in the data can make it difficult to use one data set in lieu of another, particularly during an emergency period.” (lines 443-444)

The choice of a beta-binomial model is appropriate, but the manuscript lacks detail on the hierarchical structure of the model and its implications. Were the district-level effects modeled as random effects, and if so, how were these distributed? This is an important detail that influences the interpretation of the model’s results.

**Response:** We have expanded the explanation of the beta-binomial model by moving the description from the SI Appendix into the main text (*Fitting the probability of departure* section of Materials and Methods), namely specifying the distributions from which parameters are drawn and the hierarchical structure of the model. In addition, we referred to additional details, available on the documentation page of the mobility package.

Additionally, the discussion should include the potential trade-offs involved in pooling data, such as the risk of introducing biases or obfuscating important variations that are specific to individual data sets.

**Response**: We have added the following sentence in discussion section when discussing pooling as a potential approach: “however, this may result in lower precision, and obfuscate some of the fundamental differences in types of mobility and population captured in each of the mobility datasets”. (lines 453-455)

There is also mention of districts with zero probabilities in departure indicating outliers or sparse data points. However, it should be clarified whether specific methodologies such as regularization were utilized in the modelling process in handling of such data.

**Response:** We tried several approaches, including fitting a zero-inflated model that included zero-travel from districts with zero-probability departure. However, the zero-departure districts dominated the model. As a result, we excluded the zero-departure districts from the model, instead assigning a population-level probability of departure to these districts. We have made an explicit reference to this process in the “*Fitting the probability of departure*” section of Methods and Materials (namely, “For districts that were not covered in the dataset, the model assigns a population-level probability of departure, with population mean $\tau_{p}$ drawn from a Beta distribution with population-level shape and rate parameters $\bar{\alpha}$ and $\bar{\beta}$: $\tau_{p}\sim Beta\left( 1+\bar{\alpha}, 1+ \bar{\beta} \right)$”).

The innovative aspects of this study should be highlighted more prominently, particularly in the context of the literature review. Additionally, the discussion and conclusion sections should be expanded to ensure a more logical flow and thorough exploration of the study’s implications. The conclusions drawn from the study should be clearly linked back to the objectives stated in the introduction, providing a cohesive narrative that underscores the study’s contributions to the field of infectious disease modeling.

**Response:** We have updated both the introduction and discussion to highlight additional aspects of innovation in the study.

By addressing these points, the authors can significantly enhance the manuscript’s clarity, methodological rigor, and overall impact. I recommend revising the manuscript to incorporate these critical elements and re-submitting it for further review.
