# Supplementary material for: Comparing and integrating human mobility data sources for measles transmission modeling in Zambia
Source: PLOS Glob Public Health. 2025 May 20;5(5):e0003906. doi: 10.1371/journal.pgph.0003906 (PMC12091742; doi:10.1371/journal.pgph.0003906)
Supplement: Attachment — Submitted filename: Manuscript Number-PGPH-D-24-01677R1.docx [file pgph.0003906.s016.docx]

**PLOS Global Public Health Comparing different sources of human mobility data and methods of integration: modeling measles virus transmission in Zambia.**

**--Manuscript Draft—**

First, I would like to thank **Collins Otieno Asweto** and **PLOS Global Public Health** by large, for sharing the Manuscript titled: Comparing Different Sources of Human Mobility Data and Methods of Integration: Modeling Measles Virus Transmission in Zambia.

I have reviewed the manuscript that addresses an important topic in public health particularly in Africa, in the context of infectious disease modeling. I believe it is a crucial step for accurate disease spread predictions. The use of a Bayesian framework and combining information from multiple data sets is so awesome motivation and strength of the study. However, I have a few observations where the manuscript could be improved:

1. **Title:** Despite the topic being interesting and clear, could it be possible to make it more concise; and consider the short Title: “Comparing and Integrating Human Mobility Data Sources for Measles Transmission Modeling in Zambia” as the final Title?
2. **Abstract:** You have a very nice and comprehensive Abstract but quite dense and might need only to highlight key findings and implications.
3. **Methods:** I was trying to investigate a Bayesian framework, but I didn’t get the idea well explained in the Manuscript. To help some readers like me understand, could it be possible to make it clear, and well-explained, and clarify why you chose it and its interpretations as well?
4. **Methods & Materials:** In the modeling diffusion pattern, the reason why the exponential gravity model was chosen over the others not mentioned?
5. **Result:** Under the results section, some interpretations like the travel survey data results have not been explained to inform readers why resulted in greater proportions compared to others.
6. **Discussion:** The discussion part is written in a well-detailed and thoroughness. However, focusing on the key findings and their potential impact on public health policy and practices would be critical. Expanding the already touched limitations to help address those limitations in future similar studies might also be useful.
7. **General comment**: A thorough proofreading might be important to correct minor grammatical errors and typos observed throughout the manuscript.

In general, this manuscript might be a significant and valuable contribution to the field of infectious disease modeling, and I commend it to be a strong publication in PLOS Global Public Health as long as those minor comments are addressed well.

Looking forward to seeing the revised version

Dr Adamu Gelaw
