# Supplementary material for: Comparing and integrating human mobility data sources for measles transmission modeling in Zambia
Source: PLOS Glob Public Health. 2025 May 20;5(5):e0003906. doi: 10.1371/journal.pgph.0003906 (PMC12091742; doi:10.1371/journal.pgph.0003906)
Supplement: Attachment — Submitted filename: Response to reviewers.docx [file pgph.0003906.s018.docx]

Response to reviewers

We are grateful to the reviewers for their thoughtful feedback, and have taken into account all of the suggestions. Overall, we have expanded the limitations and public health implications components of the discussion, provided more clear explanation of the Bayesian approach (both in main manuscript and in supplementary information appendix), and carried out additional proofreading.

The point-by-point responses are provided below.

Reviewer #2:

1. **Title:** Despite the topic being interesting and clear, could it be possible to make it more concise; and consider the short Title: “Comparing and Integrating Human Mobility Data Sources for Measles Transmission Modeling in Zambia” as the final Title?

**Response:** Thank you, we agree this title is more clear and have updated it accordingly.

2. **Abstract:** You have a very nice and comprehensive Abstract but quite dense and might need only to highlight key findings and implications.

**Response:** Thank you, we’ve simplified the abstract in a few locations to provide a clearer overview of the study.

3. **Methods:** I was trying to investigate a Bayesian framework, but I didn’t get the idea well explained in the Manuscript. To help some readers like me understand, could it be possible to make it clear, and well-explained, and clarify why you chose it and its interpretations as well?

**Response**: We have expanded the methods section to provide a deeper explanation of the framework. In addition, we’ve provided the Bayes’ rule as well as a figure illustrating the concept in the *SI Appendix*.

The methods section now reads: “To incorporate information on mobility from more than one data set, we used a Bayesian framework. While comprehensive description of Bayesian statistics is available elsewhere ^1^, in short, this framework relies on Bayes’ rule to incorporate information both based on the observed data (the sample) and previous information (e.g., distribution of parameters obtained from previous studies, or the prior) to obtain updated distribution for the parameters of interest (the posterior). A presentation of the Bayes’ rule as well as an illustrative figure demonstrating an example where information about prior distribution has an observable effect on the posterior distribution are provided in *SI Appendix.*

We used estimated coefficients and posterior distributions from the model fit to one data set as a prior for fitting the model to another data set. We modified the source code of the *mobility* package to include an informative prior, where population and distance parameter priors for travel between two districts were drawn from a truncated normal distribution with mean and precision set to estimates of coefficients from the diffusion model fit to another data set.

In other words, for an exponential gravity model, which was the best fit model of dispersion, the number of trips from district $i$ to district $j$, $T_{ij}$, was defined by

$$T_{ij}\sim Poisson(\mu_{ij})$$

$\mu_{ij}=\frac{\theta N_{i}^{\omega_{i}}N_{j}^{\omega_{j}}}{e^{d_{ij}/\delta}}$,

where $T_{ij}$ is the number of trips from district $i$ to district $j$, with $i$ and $j$ distinct; $N_{i}$ is the population size of the origin district; $N_{j}$ is the population size of the destination district; and $d_{ij}$ is the distance between the centroids of the two districts, in kilometers. The $\omega_{i}$ and $\omega_{j}$ parameters modify the contribution of origin and destination population sizes, respectively, while $\delta$ is the distance deterrent parameter, modifying the extent to which distance between origin and destination reduces the probability of travel, while $\theta$ is the scaling parameter. To combine information from two datasets, we used information on the distribution of fitted parameters from the first dataset to generate the following priors:

$$\omega_{1}\sim Truncnorm(mean(\hat{\omega_{1}}), sd(\hat{\omega_{1}}))$$

$$\omega_{2}\sim Truncnorm(mean(\hat{\omega_{2}}), sd(\hat{\omega_{2}})$$

$$\delta\sim Truncnorm(mean\left( \hat{\delta} \right), sd\left( \hat{\delta} \right))$$

Where $mean\left( \hat{\varphi} \right)$ and $sd\left( \hat{\varphi} \right)$ are the mean and standard deviation of the distribution for the fitted $\varphi$ parameter obtained using data from the first source.

Then, data from the second source was used as sample, and a posterior is obtained for each of the parameters.”

4. **Methods & Materials:** In the modeling diffusion pattern, the reason why the exponential gravity model was chosen over the others not mentioned?

**Response:** We’ve revised the text to include mention of the goodness of fit metrics used to select the best fit model (exponential gravity model). The corresponding section of methods now reads: “We first fit gravity models with different structures and selected a best-fit model for each data set, assessing model fit using goodness of fit metrics (deviance information criterion, root-mean-square error, mean absolute percentage error, and R^2^) and summary plots for the fitted models. Key summaries of metrics for gravity models are presented in *SI Appendix*.” The *SI Appendix* includes plots of model fits, as well as summaries of goodness of fit statistics.

5. **Result:** Under the results section, some interpretations like the travel survey data results have not been explained to inform readers why resulted in greater proportions compared to others.

**Response:** We have added the following sentence to the discussion (bolded), and believe this addresses the Reviewer’s comment. We find that the discussion section is a more appropriate section to provide conjectured explanations, rather than the Results.

**“**Previous studies have shown that survey-based reporting of travel may differ by trip purpose ^2^, and may underreport frequency of travel while over-reporting long-distance trips ^3^. **The latter could explain why the distance deterrent coefficient was much smaller in the travel survey than in mobile phone and Facebook data, which could in turn explain why use of travel survey findings in simulations of measles transmission resulted in introduction events across the country.** If individuals are less likely to report shorter, more routine trips, we are likely to overestimate long-distance travel, resulting in an inflated probability of departure. Furthermore, the relatively small sample size of surveys may result in capturing of the high-volume travel routes, but is less likely to capture smaller-frequency connections between origin and destination districts.”

6. **Discussion:** The discussion part is written in a well-detailed and thoroughness. However, focusing on the key findings and their potential impact on public health policy and practices would be critical. Expanding the already touched limitations to help address those limitations in future similar studies might also be useful.

**Response**: Thank you, we’ve provided additional insight focusing on the impact on public health policy and practices, with specific example of supplementary immunization activities in Zambia. In addition, we’ve expanded upon the limitations, with special focus on travel survey and Facebook data, and provided suggestions for future research.

7. **General comment**: A thorough proofreading might be important to correct minor grammatical errors and typos observed throughout the manuscript.

**Response**: Thank you, we’ve carried out additional proofreading and addressed the errors that we could find.

Reviewer #3: A thorough proofreading might be important to correct minor grammatical errors and typos observed throughout the manuscript

**Response**: Thank you, we’ve carried out additional proofreading and addressed the errors that we could find.

Reviewer #4:

Some sections of the methods could be clarified or expanded slightly.

**Response:** We expanded the Methods section to include a more in-depth description of the weighting approach used to correct for biases of mobile phone and Facebook data, and also provided a more explicit description of how Bayesian framework is used to integrate data from two data sets. More details are provided in the specific comments below.

Additional discussion of limitations would strengthen the paper.

**Response:** We have expanded the discussion section to include additional limitations of the Facebook and travel surveys specifically, and also expanded the recommendations section.

A few minor grammatical and formatting issues to address.

**Response**: Thank you, we’ve carried out additional proofreading and addressed the errors that we could find.

Methods: The weighting approach for mobile phone and Facebook data could be explained in slightly more detail in the main text.

**Response:** We have moved the corresponding description of weighting from the supplementary information appendix to main text. Namely, the Methods section now includes the following: “For example, we used the following approach to calculate the weighted mobile phone probability of departure:

$${P\left( t=1 \right)}_{wMP}={P\left( t=1 \right)}_{MP}P\left( mp=1 \right)_{DHS}+P\left( t=1 \right)_{MP}\frac{P\left( t=1 | mp=0 \right)_{DHS}}{P\left( t=1 | mp=1 \right)_{DHS}}P\left( mp=0 \right)_{DHS}$$

Where $P\left( t=1 \right)$ is the probability of departure; $P\left( mp=1 \right)$ is the probability of owning a mobile phone; $P\left( mp=0 \right)$ is the probability of not owning a mobile phone; and $P\left( t=1|mp=i \right)$ is the probability of departure conditional on mobile phone ownership. Subscripts indicate the data set, where $MP$ is mobile phone data, and $wMP$ is weighted mobile phone data. Note that we only collected mobile phone data from a single operator but are taking the simplifying assumption that the ratio $\frac{{P\left( t=1 | mp=0 \right)}_{DHS}}{P\left( t=1 | mp=1 \right)_{DHS}}$ captures the relationship between the travel of Zamtel users and non-Zamtel users. The same approach was used to weight the Facebook data using Internet use rather than mobile phone ownership.”

Consider adding a brief explanation of why measles was chosen as the case study disease.

**Response**: Thank you for your suggestion. We have added the following sentence in the introduction to explain why measles was selected: “Measles was selected as an illustrative example given Zambia’s remarkable progress towards elimination since the introduction of nationwide supplementary immunization activities in 2003 ^4^, and potential importance of identifying areas at high risk for introduction events to better target nationwide vaccination strategy, including subnational campaigns.”

Consider adding confidence intervals to some of the key numerical results.

**Response:** We provided confidence intervals for key results like cumulative infections in Table S3, as well as in the figures. For probability of travel, we find that IQR is a more appropriate measure.

Discussion:
Further discussing limitations of the study and datasets used.
Expanding on potential applications of the integrated mobility estimates.
Suggesting future research directions.

**Response:** We have expanded the discussion section to include stronger focus on public health significance of the findings, future research directions, and importance of integrating information across multiple mobility data sets.

Minor Issues:
A few grammatical errors and typos to correct (e.g., "ninemonth" should be "nine-month").

**Response**: Thank you, we’ve carried out additional proofreading and addressed the errors that we could find.

Ensure consistent formatting of headings throughout.

**Response:** Thank you, we’ve updated the formatting for consistency.

Double-check all in-text citations match the reference list.

**Response**: Thank you, we verified that these are ok.

Reviewer #5: 
Some sections, particularly those describing the Bayesian modeling approach and simulation, could benefit from clearer explanations or additional detail for readers who may not be familiar with the technical methods used.

**Response:** Thank you, we have added more detail describing Bayesian modeling approach (as provided below). In addition, details about the transmission model and simulation are made available in the *SI Appendix*.

Streamlining and reducing redundancy in the introduction and discussion sections would improve readability.

**Response:** We have checked that redundancy in introduction and discussion sections are minimal.

More detail on the parameterization of the metapopulation model and sensitivity analyses would help ensure reproducibility.

**Response**: We have made available all parameter values along with more detailed description of the metapopulation model in the Supplementary information appendix (“Transmission model specification”). The only difference in sensitivity analysis is the distribution of initial seeds of infection, with 5 cases introduced in each of the two districts of interest, Choma and Ndola. This is described as follows: “As sensitivity analysis, we conducted simulations of measles virus dynamics following the introduction of 5 cases each in Ndola District in northern Zambia and Choma District in southern Zambia. We used 5 cases in each of the two districts to seed the same number of total infections as in the main scenario (10) but distributed between the two districts due to their lower connectivity compared to Lusaka District.”

To ensure reproducibility, we have also made all code available on Github.

For example, providing more specifics on how the priors were set for the Bayesian model and how they influence the outcomes would be beneficial. Including a visual representation of the Bayesian framework and the data integration process could enhance the clarity of the methods.

**Response**: We added the following description to the main text: “While comprehensive description of Bayesian statistics is available elsewhere ^1^, in short, this framework relies on Bayes’ rule to incorporate information both based on the observed data (the sample) and previous information (e.g., distribution of parameters obtained from previous studies, or the prior) to obtain updated distribution for the parameters of interest (the posterior). A presentation of the Bayes’ rule as well as an illustrative figure demonstrating an example where information about prior distribution has an observable effect on the posterior distribution are provided in SI Appendix.”

In addition, we have moved details on the Bayesian framework as applied to this analysis from supplementary information document to the main text. The Methods section now includes the following description of how information from two data sets was integrated in the framework: “In other words, for an exponential gravity model, which was the best fit model of dispersion, the number of trips from district $i$ to district $j$, $T_{ij}$, is defined by

$$T_{ij}\sim Poisson(\mu_{ij})$$

$\mu_{ij}=\frac{\theta N_{i}^{\omega_{i}}N_{j}^{\omega_{j}}}{e^{d_{ij}/\delta}}$,

where $T_{ij}$ is the number of trips from district $i$ to district $j$, with $i$ and $j$ distinct; $N_{i}$ is the population size of the origin district; $N_{j}$ is the population size of the destination district; and $d_{ij}$ is the distance between the centroids of the two districts, in kilometers. The $\omega_{i}$ and $\omega_{j}$ parameters modify the contribution of origin and destination population sizes, respectively, while $\delta$ is the distance deterrent parameter, modifying the extent to which distance between origin and destination reduces the probability of travel, while $\theta$ is the scaling parameter. To combine information from two datasets, we use information on the distribution of fitted parameters from the first dataset to generate the following priors:

$$\omega_{1}\sim Truncnorm(mean(\hat{\omega_{1}}), sd(\hat{\omega_{1}}))$$

$$\omega_{2}\sim Truncnorm(mean(\hat{\omega_{2}}), sd(\hat{\omega_{2}})$$

$$\delta\sim Truncnorm(mean\left( \hat{\delta} \right), sd\left( \hat{\delta} \right))$$

Where $mean\left( \hat{\varphi} \right)$ and $sd\left( \hat{\varphi} \right)$ are the mean and standard deviation of the distribution for the fitted $\varphi$ parameter obtained using data from the first source.

Then, data from the second source is used as sample, and a posterior is obtained for each of the parameters.”

While the manuscript discusses limitations related to sampling biases, especially regarding mobile phone and Facebook data, it could be further strengthened by explicitly addressing how these biases might affect the generalizability of the findings. For instance, the demographic differences in mobile phone ownership (age, gender, socio-economic status) could significantly impact the conclusions drawn.

**Response**: We’ve expanded the discussion section to make more explicit statements on how some of the biases are likely to affect the findings (e.g., Facebook data likely to under-report lower-volume routes of travel; travel surveys likely to overreport long-distance travel), and also included a call for correcting for sampling biases when possible – although this is challenging given that few data sources, to our knowledge, make the necessary data available.

A more in-depth discussion on the potential biases introduced by using aggregated Facebook data, especially during the COVID-19 pandemic, and how this might influence mobility patterns is warranted. Although the ethical considerations regarding data use are addressed, emphasizing how data privacy concerns were mitigated, particularly with Facebook and mobile phone data, would be important for transparency.

**Response**: We have briefly touched upon this in the discussion section. Namely, we added a paragraph that reads, “Facebook data are similarly subject to sampling biases as well as issues with accuracy of location. Increasing use of multiple Global Navigation Satellite Systems as opposed to GPS provides a promising direction for increasing accuracy of geo-location on a large scale (46). Individuals that do not use a smartphone, do not have Facebook application installed, or choose not to opt in to location sharing on their application are excluded from the data set. Furthermore, to protect privacy and confidentiality of its users, the data set omits information on routes between destination and origin tiles that have less than ten users reporting travel; as a result, Facebook data are likely to bias models towards higher-flow routes.”

The limitations are well-discussed, particularly regarding the biases inherent in different data sources and the challenges of integrating them. However, a deeper exploration of how these limitations might affect public health policy decisions based on the findings would provide more context for the study's real-world applications.
**Response:** We have focused the discussion to more clearly emphasize the importance of relying on multiple sources of mobility when they are available. Up to this date, we have not observed this to be the case; namely, a systematic review focusing on use of mobility data in quantifying transmission of SARS-CoV-2 found that, despite availability of multiple sources of mobility data, generally, only a single data set was used by researchers in their studies ^5^. Our study highlights the importance of carefully selecting the data source, and bringing together data from multiple sources when possible.

**References**

1.	van de Schoot, R. et al. *Child Dev.* **85**, 842–860 [2014].

2.	Forrest, T.L. & Pearson, D.F. *Transp. Res. Rec.* **1917**, 63–71 [2005].

3.	Stopher, P., FitzGerald, C. & Xu, M. *Transportation* **34**, 723–741 [2007].

4.	World Health Organization [2022].at <https://www.who.int/data/gho/data/indicators/indicator-details/GHO/measles---number-of-reported-cases>

5.	Kostandova, N. et al. *Nat. Commun.* **15**, 10504 [2024].
