# Supplementary material for: Comparing and integrating human mobility data sources for measles transmission modeling in Zambia
Source: PLOS Glob Public Health. 2025 May 20;5(5):e0003906. doi: 10.1371/journal.pgph.0003906 (PMC12091742; doi:10.1371/journal.pgph.0003906)
Supplement: Attachment — Submitted filename: response_to_reviewers_auresp_3.docx [file pgph.0003906.s019.docx]

Response to editors

We are thankful for the editorial team for the issues raised. We have addressed them as noted below.

1. We have noticed that you have uploaded Supporting Information files, but you have not included a list of legends. Please add a full list of legends for your Supporting Information files before or after the references list.

Response: We have included Supporting Information Captions right after the References section.

2. Some material included in your submission may be copyrighted. According to PLOS’s copyright policy, authors who use figures or other material (e.g., graphics, clipart, maps) from another author or copyright holder must demonstrate or obtain permission to publish this material under the Creative Commons Attribution 4.0 International (CC BY 4.0) License used by PLOS journals. Please closely review the details of PLOS’s copyright requirements here: PLOS Licenses and Copyright. If you need to request permissions from a copyright holder, you may use PLOS's Copyright Content Permission form.

Response: The base map used was made available by OCHA under the Creative Commons Attribution for Intergovernmental Organisations license (Source: <https://data.humdata.org/dataset/cod-ab-zmb>, description of the licenses: <https://data.humdata.org/faqs/licenses>). Notably, “Under the CC BY-IGO license, you are free to share (copy and redistribute the material in any medium or format) and or adapt (remix, transform, and build upon the material) for any purpose, even commercially. The licensor cannot revoke these freedoms as long as you follow the license terms. The license terms are that you must give appropriate credit, provide a link to the license, and indicate if changes were made. You may do so in any reasonable manner, but not in any way that suggests the licensor endorses you or your use. Additionally, you may not apply legal terms or technological measures that legally restrict others from doing anything the license permits. When the Licensor is an intergovernmental organization, disputes will be resolved by mediation and arbitration unless otherwise agreed.” We believe this is consistent with the PLOS copyright requirements.

We have added the following description in the Fig 1 legend: Maps were created in R, using [base layer shapefile provided by OCHA](https://data.humdata.org/dataset/01c71cfd-32ff-4a32-a1dd-5909702c12db/resource/4175df29-f71b-479a-9a03-ca2b54af51c0/download/zmb_adm_dmmu_20201124_shp.zip) under Creative Commons Attribution for Intergovernmental Organisations license [37].
