# Supplementary material for: Comparing and integrating human mobility data sources for measles transmission modeling in Zambia
Source: PLOS Glob Public Health. 2025 May 20;5(5):e0003906. doi: 10.1371/journal.pgph.0003906 (PMC12091742; doi:10.1371/journal.pgph.0003906)
Supplement: Attachment — Submitted filename: response to reviewers_20250326.docx [file pgph.0003906.s020.docx]

Response to editors

We are thankful to the reviewers for the feedback and suggestions. We expanded the discussion section to delve deeper into the application of findings to inform public policy and the potential directions for incorporating other mobility data sources. In addition, we updated to abstract to read more clearly, and updated one of the citations (Reference 18) from a pre-print to a peer-reviewed reference as it was published.

We have addressed specific comments as noted below.

Reviewer #1: The manuscript "Comparing and integrating human mobility data sources for measles transmission modeling in Zambia" has met all the requirements to be considered acceptable for publication. I would recommend the manuscript for PLOS Global Public Health publication.

Response: Thank you!

Reviewer #2: Thank you for adding the legends as well as clarifying the License. Humdata states all data is CC BY 4 unless otherwise noted, which in my opinion should satisfy PLOS requirements.

Response: Thank you! We agree this should satisfy the requirements.

Reviewer #4: Recommendations for Publication
Clarify Data Integration Challenges: Provide more detailed explanations of the technical challenges encountered during data integration and how they were addressed.
Response: We expanded the discussion section to include more explicit explanation of how some of the technical challenges were approached. For example, we added the following text (in itallics): “The range in spatial coverage of each data set made it difficult to perform systematic comparisons of how they differed and methods to combine data sets. *While we modeled mobility using available data, and used predicted mobility for geographical areas where data were missing, this relied on the assumption that mobility data were missing at random.*”

Furthermore, we added the following sentences to underscore how we approached the issue of sampling bias in mobile phone data and Facebook data (in italics) “Facebook data are similarly subject to sampling biases as well as issues with accuracy of location. Increasing use of multiple Global Navigation Satellite Systems as opposed to GPS provides a promising direction for increasing accuracy of geo-location on a large scale [46]. Individuals that do not use a smartphone, do not have Facebook application installed, or choose not to opt in to location sharing on their application are excluded from the data set. *To mitigate the issue of sampling bias in mobile phone and Facebook data, we used information available in DHS to adjust mobility estimates, accounting for heterogeneity in travel outside district for individuals with and without mobile phones, and reporting or not reporting Internet use in the last 12 months. However, as these questions capture a different facet of mobility and a different time period, there likely remains residual bias.*”

We also identified some of the persistent challenges in the discussion section that require further investigation and approaches that are not explored in this study. Namely, we discuss the challenge of using Bayesian framework to integrate data sets where one data set has much larger sample size and hence dominates the posterior. We provide an interpretation and suggestions on how to approach this issue, namely using external information to determine how informative to make each dataset, or using pooling when such information is not available. However, while we carry out pooling in our study, we acknowledge that this results in lower precision and does not take into account that datasets used capture fundamentally different type of mobility data.

Expand Discussion on Public Health Implications: Further elaborate on how the findings can inform public health policy and disease control strategies beyond Zambia.
Response: We added the following to the discussion: “While this study was carried out in Zambia, the implications of these findings are applicable to other settings and phases of public health response. Since mobility data is increasingly being used to inform decision making before, during, and after a disease outbreak, understanding how various data sets can be used to produce realistic estimates of spatial pathogen dynamics is needed. The choice of data set can impact estimates of introduction risk which would ultimately influence the scale and focus of public health interventions.”

Consider Additional Data Sources: Discuss potential future directions for incorporating other mobility data sources, such as GPS loggers or transportation flows, to enhance model accuracy.

Response: We have updated the final paragraph of discussion to read as follows (in italics), while acknowledging that these data sources are also not free of bias. “*Increased availability of mobility data through sources like GPS trackers, mobile phone, social media, satellite data, retail and commercial data, and others offer a unique opportunity to refine and produce more accurate estimates of mobility. However, further research on ways to correct for sampling and information biases inherent in each of the data sets, and implications of these corrections on public health inference, is necessary.”*

Overall, the article contributes significantly to the field of infectious disease modeling by highlighting the importance of integrating diverse data sources to improve the accuracy of mobility estimates and disease transmission dynamics. With minor revisions to address the suggested areas, the article would be well-suited for publication in PLOS Global Public Health.

Response: Thank you!

Reviewer #6: The concept of this manuscript is a bit hard to grasp. If it were explained in simpler terms, it would be much easier for general readers to understand the idea.

Response: We have now updated the abstract to help convey the general idea of the manuscript.

Quantifying population mobility is crucial in developing accurate models of infectious disease dynamics. Increasingly, multiple data sources are available to describe individual and population mobility in a single location; however, there are no methods to systematically integrate these data. Combining information from these data sets may be valuable and help mitigate inherent biases in each data set due to sampling, censoring, and recall. We examined four commonly used data sources (mobile phone records, travel survey, Demographic and Health Survey, and Facebook location information) to quantify subnational travel patterns in Zambia. First, we explored summary metrics of mobility from each data set. Estimates of the probability of a trip and location of travel varied across data sets, with some data quantifying twice the frequency of travel as others. Then, we developed a departure-diffusion model that is able to produce a single estimate of travel by combining these data sets. When multi-data set models included mobile phone records, this data source dominated estimates given the broader spatial coverage. We then used a metapopulation model to simulate a measles outbreak to identify how these different data sets and models would impact estimates of the spatial spread of a highly infectious disease. We found that using travel survey data to parameterize mobility resulted in the introduction of cases in 98% of districts compared to less than 50% when mobile phone data or Facebook data were used. This study highlights the need for methods to integrate multiple data sets to improve validity of mobility estimates and resultant infectious disease transmission dynamics.
